# Frailty detection among primary care older patients through the Primary Care Frailty Index (PC-FI)

**DOI:** 10.1038/s41598-023-30350-3

**Published:** 2023-03-02

**Authors:** Davide Liborio Vetrano, Alberto Zucchelli, Graziano Onder, Laura Fratiglioni, Amaia Calderón-Larrañaga, Alessandra Marengoni, Ettore Marconi, Iacopo Cricelli, Pierangelo Lora Aprile, Roberto Bernabei, Claudio Cricelli, Francesco Lapi

**Affiliations:** 1 Aging Research Center, Department of Neurobiology, Care Sciences and Society, Karolinska Institutet and Stockholm University, Tomtebodavägen 18 A, 10th Floor, Solna, 171 65 Stockholm, Sweden; 2 Stockholm Gerontology Research Center, Stockholm, Sweden; 3 Department of Clinical and Experimental Sciences, University of Brescia, Brescia, Italy; 4 Fondazione Policlinico Gemelli IRCCS, Rome, Italy; 5 Università Cattolica del Sacro Cuore, Rome, Italy; 6 Health Search, Florence, Italy; 7 Italian College of General Practitioners and Primary Care, Florence, Italy

**Keywords:** Health care, Diagnosis, Geriatrics, Prognosis, Public health

## Abstract

The prompt identification of frailty in primary care is the first step to offer personalized care to older individuals. We aimed to detect and quantify frailty among primary care older patients, by developing and validating a primary care frailty index (PC-FI) based on routinely collected health records and providing sex-specific frailty charts. The PC-FI was developed using data from 308,280 primary care patients ≥ 60 years old part of the Health Search Database (HSD) in Italy (baseline 2013–2019) and validated in the Swedish National Study on Aging and Care in Kungsholmen (SNAC-K; baseline 2001–2004), a well-characterized population-based cohort including 3363 individuals ≥ 60 years old. Potential health deficits part of the PC-FI were identified through ICD-9, ATC, and exemption codes and selected through an optimization algorithm (i.e., genetic algorithm), using all-cause mortality as the main outcome for the PC-FI development. The PC-FI association at 1, 3 and 5 years, and discriminative ability for mortality and hospitalization were tested in Cox models. The convergent validity with frailty-related measures was verified in SNAC-K. The following cut-offs were used to define absent, mild, moderate and severe frailty: < 0.07, 0.07–0.14, 0.14–0.21, and ≥ 0.21. Mean age of HSD and SNAC-K participants was 71.0 years (55.4% females). The PC-FI included 25 health deficits and showed an independent association with mortality (hazard ratio range 2.03–2.27; *p* < 0.05) and hospitalization (hazard ratio range 1.25–1.64; *p* < 0.05) and a fair-to-good discriminative ability (c-statistics range 0.74–0.84 for mortality and 0.59–0.69 for hospitalization). In HSD 34.2%, 10.9% and 3.8% were deemed mildly, moderately, and severely frail, respectively. In the SNAC-K cohort, the associations between PC-FI and mortality and hospitalization were stronger than in the HSD and PC-FI scores were associated with physical frailty (odds ratio 4.25 for each 0.1 increase; *p* < 0.05; area under the curve 0.84), poor physical performance, disability, injurious falls, and dementia. Almost 15% of primary care patients ≥ 60 years old are affected by moderate or severe frailty in Italy. We propose a reliable, automated, and easily implementable frailty index that can be used to screen the primary care population for frailty.

## Introduction

In our aging societies, most healthcare expenditure is disproportionally—and often inefficiently—absorbed by older adults with frailty^1,2^. Frailty is understood as a clinical syndrome characterized by depleted physiological reserve across different organs and systems that exposes affected individuals to the worst consequences of clinical acute episodes, such as infections, cardiovascular events, and injuries^3–5^. Frailty is a major obstacle to the accomplishment of longer and healthier lives, even more than diseases, as it has been associated with increased risk of disability, dementia, hospitalizations, institutionalization, and death^6–12^. In accordance with a recent meta-analysis, the global prevalence of frailty among community-dwelling individuals 50 + ranges between 11 and 26%, depending on which frailty definition is used^13^. In this scenario, the prompt identification of seniors with frailty and complex care needs and at a higher risk of developing poor health outcomes may contribute to guarantee high and sustainable care standards for an ever-increasing number of older adults^9^.

An effective and timely management of older people with frailty depends on the availability of accurate multidimensional assessment tools to identify this syndrome. The implementation of such instruments on a large scale would maximize the benefits at the population level while ensuring equal healthcare provision^9^. Primary care has been suggested to be the ideal care setting to screen patients for frailty^14–17^. Primary care services are the gatekeepers of our healthcare systems; general practitioners (GPs) have an extensive knowledge of patients’ clinical and life-course biographies. Importantly, electronic primary care health records represent a unique source of comprehensive and up to date patient information, which could potentially be exploited to screen for frailty at the population level^18^.

During the last two decades, several conceptual frameworks have been proposed to define and assess frailty. However, most existing assessment tools require specific training, instruments, space, and, most importantly, extended time, which is seldom compatible with primary care routines^19,20^. For this reason, of the several available frailty assessment tools, the frailty index (FI)—based on the principle that we accumulate health deficits with age—appears particularly suitable for the primary care setting. The FI is calculated as the ratio between the number of health deficits (e.g., diseases, symptoms, signs, disabilities) affecting a person and the total number of deficits considered by the assessor. The result is a number between zero and one, which is easily interpretable and that may be further categorized according to frailty severity^18,20^. The FI may be used to stratify patient’s according to their risk and to identify those in need of special care, as suggested by the Italian and UK guidelines for the management of patients with multimorbidity^21,22^. Of note, the FI can be easily determined using information derived from routinely collected primary care data, as in the case of the UK electronic FI (eFI), so allowing an automated calculation by GP’s software. The UK eFI has shown fair-to-good discriminative ability in the prediction of mortality and hospitalizations, and its utilization in primary care is recommended by the UK National Health System (NHS)^18^. Importantly, the deficits included in the UK eFI have been selected solely based on clinical consensus. There are reasonable grounds to hypothesize that a data-driven approach to the selection of deficits could lead to the construction of a more reliable and highly predictive FI, by optimizing the utilization of the available information^23^.

In this study, we aimed to detect frailty among the primary care older population, by developing and validating a FI based on Italian routinely collected primary care data, employing a previously developed data-driven methodology for the selection of deficits. Frailty charts with mortality nomograms, separate for women and men, were also built. Moreover, we aimed to test the convergent validity of the newly proposed FI against a validated measure of physical frailty and several frailty-related outcomes including disability, falls, and dementia.

## Methods

### Development and internal validation population

We developed our FI using data from the Health Search Database (HSD), an Italian primary care database^24,25^. We will refer to our FI as the HS-Primary Care FI (hereinafter PC-FI) from now on, to avoid confusion with other FIs. Since 1998, the HSD has collected data from a network of about 800 Italian GPs, who collect and register patients’ routine clinical information. The network of participating GPs is evenly distributed across Italy, covering a population of more than one million primary care patients. The HSD contains information on demographics, clinical diagnoses, drug prescriptions and diagnostic tests, specialist referrals, hospital admissions, and death. Diseases are classified in accordance with the International Classification of Diseases 9^th^ Revision Clinical Modification (ICD-9CM), drug prescriptions according to the Anatomical Therapeutic Chemical (ATC) system, economic exemptions and diagnostics tests and referrals according to codes issued by the Italian Ministry of Health. For the purposes of our study, we analyzed a random cohort of 308,280 individuals 60 + years old, followed up by the same GP for at least 5 years (median follow-up time 6.9 years) if death did not occur earlier, across a timeframe from 1 January 2013 to 31 December 2019. For this study, the entry date was 1 January 2013 for those that turned 60 before that date or their 60th birthday for the others. Data were randomly split into a training dataset (N = 184,968; 60%) employed for the construction of the FI, and a testing dataset (N = 123,312; 40%) used for the internal validation. The characteristics of the two subsamples were identical as shown in Table S1. In accordance with the Italian legislation, the permission to use anonymized electronic healthcare records is granted for observational epidemiological research as the present one. All methods were performed in accordance with the relevant guidelines and regulations of international Code of Conduct. The study was reported in keeping with the transparent reporting of a multivariable prediction model for individual prognosis or diagnosis (TRIPOD) recommendations.

### External validation population

For the external validation of the PC-FI, we used data from the Swedish National Study on Aging and Care in Kungsholmen (SNAC-K), an ongoing population-based study started in 2001 and involving individuals 60 + years old living in the Kungsholmen district in central Stockholm, Sweden. Thirteen years of follow-up were used for the present study. At baseline (i.e., 2001–2003), 3363 persons (73.3% response rate) underwent a comprehensive assessment using standard questionnaires administered by trained nurses, medical examinations, and instrumental and blood tests to retrieve information about their demographics, and clinical and functional status. Individuals’ information was also linked with the Swedish National Patient Register, Stockholm Regional Outpatient Register and the Swedish Death Register. An in-depth description of the data collection protocols is available elsewhere^26^. Informed consent was obtained from each participant, or from a proxy in case of cognitive impairment. The study was approved by the Regional Ethical Review Board in Stockholm.

### Deficit identification

Individual-level characteristics describing their health status across different organ and systems were considered as potential deficits. Overall, 101 potential deficits were identified in the HSD, based on a clinical appraisal of the information available in the dataset and looking backward to the whole patients’ available health record (Table S2): (a) 64 groups of chronic diseases; (b) 17 signs and symptoms reported in the six months prior to study entry date, (c) 11 related to healthcare utilization in the six months prior to study entry date (e.g., number of GP visits, emergency department admissions, or hospitalizations), (d) six related to acute conditions or drug prescriptions in the six months prior to study entry date (e.g., infectious diseases, oxygen prescription), and (e) three addressing functional or financial difficulties. Chronic diseases were defined based on a previously published list of 60 categories of conditions proposed by Calderón-Larrañaga et al. and identified by means of their ICD-9CM codes^27^. Signs, symptoms, and acute conditions were also identified through ICD-9CM codes. In the HSD, recently prescribed drugs, diagnostic tests, and specialist referrals are identified by means of the ATC and Italian Ministry of Health codes, respectively. Several exonerations, as registered by GPs through specific codes, were used to identify disability and economical vulnerabilities (i.e., financial exonerations for drugs, or financial exonerations for patients with disability). All deficits were coded to range between 0 and 1. Five potential deficits had 3 levels (i.e., 0, 0.5 and 1) and the remaining included two values (i.e., 0 and 1; Table S2).

In SNAC-K, only those deficits selected for the PC-FI were computed for the external validation. Information on these deficits was obtained through comprehensive interviews, physical examinations, and register data, as reported in Table S3. Diseases were coded using ICD-10 codes, which were mapped to ICD-9 codes via official mapping sheets.

### Frailty index construction: the genetic algorithm

To select the deficits to be included in the PC-FI, we employed a *genetic algorithm*. A *genetic algorithm* is an optimization algorithm employed to find near-optimal solutions for problems where it is not computationally feasible to evaluate all possible combinations of elements (e.g., the deficits to include in a frailty index). An in-depth explanation of this methodology applied to frailty indices is available elsewhere and in the Supplementary methods^23^. Shortly, a *genetic algorithm* iteratively tests the discriminative ability of a group of frailty indices in the prediction of mortality, selects those exhibiting better performances, and randomly combine them, creating new frailty indices. These newly created frailty indices replace those showing worse predictive performances. At the beginning, the frailty indices evaluated by the *genetic algorithm* are randomly created and their predictive performance is low, but iteration after iteration, frailty indices exhibiting better and better predictive performances are selected by the algorithm. The algorithm stops when a certain number of iterations is run or when it fails to find a better frailty index. In this study, the number of frailty indices evaluated in each iteration was 1500. The predictive performance for mortality—used as the only outcome to assess models’ fitness—was calculated as the average c-statistic obtained through unadjusted Cox regression models in the whole sample and in specific sex- (male and female), age- (younger and older than 71 years—the median age), and geographic area- (Northern, Central, and Southern Italy) subsamples. All-cause mortality over the whole follow up period was used as outcome.

We ran the *genetic algorithm* 50 times: in each instance, the training sample was based on different groups of 10,000 randomly chosen participants among those included in the training dataset. To explore the relative importance of each deficit, we counted the number of times each deficit was included in the FI that exhibited the highest average c-statistic in its iteration. The final and best PC-FI was the one conformed by all of the most important deficits and showed the highest average c-statistic in the whole training dataset.

Four cut-offs were identified to stratify the study population in the following categories: “fit”, “mildly frail”, “moderately frail”, “severely frail”. We identified such categories by creating four equally spaced PC-FI intervals using the 99^th^ percentile of PC-FI as the upper limit^18^. The 99th percentile of the PC-FI was 0.28 and the intervals used to categorize frailty were: < 0.07, 0.07 to < 0.14, 0.14 to < 0.21, and ≥ 0.21.

For a secondary analysis, the eFI as proposed by Clegg et al. was calculated in the HSD^18^.

### Outcomes

Mortality rate over 5 years was the outcome used to train our algorithm during the construction of the FI in HSD. Hospitalization was also tested as an alternative outcome. In the HSD, information on 5-year mortality and first hospitalization was retrieved from the GPs health records, where specific codes and the date of occurrence are used to register such events in their software.

For the external validation of the FI in the SNAC-K cohort, hospitalization and mortality information was retrieved from the Swedish National Patient Register and the Swedish Death Register, respectively. Furthermore, to test the convergent validity of our FI, several clinical and functional outcomes—both cross-sectional and longitudinal—were collected from the SNAC-K population^28–30^. Disability was defined as the presence of at least one impairment in the activities of daily living (ADL; grooming/personal hygiene, dressing, toileting/continence, transferring/ambulating, and eating) and instrumental ADL (IADL; managing finances, managing transportation, shopping, meal preparation, housecleaning, laundry, use of telephone, managing medications). Physical frailty, is another commonly used conceptual model to assess frailty, and was evaluated according to the frailty phenotype proposed by Fried L. et al*.*, using a cut-off of at least three out of five frailty criteria (i.e., unintentional weight loss, low energy expenditure, self-reported exhaustion, slow gait speed and weak grip strength)^19^. Gait speed was timed as participants walked 6 m, or 2.4 m for those who considered themselves slow walkers. Slow gait speed was defined as walking < 0.8 m/s. Unintentional weight loss was defined as the loss of at least one kg within the last three months. Those exercising three times per month or less were said to have low energy expenditure. Self-reported exhaustion was defined as reported fatigue within the last three months. Grip strength (Newtons) was measured in both hands with an electronic dynamometer (Grippit®), using the strongest value of the two. Weak grip strength was classified as the lowest 20% of participants, adjusted by sex and body mass index. The chair stand test was performed by asking participants to fold their arms across their chest and stand up from a seated position five times consecutively as quickly as possible, and the results were expressed in seconds. A test lasting > 17 s indicated poor performance. Dementia diagnosis was based on the DSM-IV criteria. Injurious falls were defined as any fall requiring medical attention. Data on injurious falls during the study period were obtained from diagnoses made at the patients’ hospital discharge and identified through the ICD-10 codes W00 to W19.

### Statistical analysis

The characteristics of the study populations were described using counts and proportions, or medians and interquartile ranges, as appropriate. Group differences were investigated using the chi-squared test or the Mann–Whitney test, as appropriate. The associations between the PC-FI and the outcomes (i.e., all-cause mortality and hospitalization) were investigated using unadjusted and adjusted Cox regression models. Death and/or hospitalization (as appropriate), and end of the 5 years follow up were considered censoring events. Time-to-last follow up was further truncated at 1, 3 and 5 years. The proportional hazard assumption (tested according the Schoenfeld residuals test) was ascertained in all analyses. To investigate the discriminative ability of the PC-FI and single deficits in the prediction of mortality in HSD and SNAC-K, we calculated the c-statistic of unadjusted Cox regression models. The association between the four PC-FI categories and mortality was investigated using Cox regression models. Psychometric properties of the PC-FI (i.e., accuracy, sensitivity, specificity, positive predictive value, negative predictive value, positive likelihood ratio and negative likelihood ratio) have been obtained. In the external validation cohort (i.e., SNAC-K), the association of the PC-FI with physical frailty and other outcomes was examined using unadjusted and adjusted logistic regression models. The area under the curve from non-parametric ROC analyses was used to assess the discriminative ability of the PC-FI, based on the unadjusted logistic regression models.

Finally, the percentiles of PC-FI were calculated and graphically represented by age in the whole HSD, stratified by sex. In the same graphs, the estimated risk of death at 5 years for different combinations of PC-FI and chronological age was presented through heatmaps in nomograms. The probability of 3-year mortality was estimated using age and PC-FI as predictors in two second-order polynomial logistic regression models (one for men and one for women). A 5-year mortality predicted probability of 50% was used as a midpoint for the color scale.

### Role of the funding source

The funders of this work did not play any role in all the phases of the research.

## Results

Table 1 shows the characteristics of the HSD and SNAC-K populations. In the HSD, the median age was 71 years old (interquartile range [IQR] = 65–78; range 60–108), and 55.4% of participants were female. More than 10% of the HSD study population had a disability financial exoneration, and 88.3% were affected by multimorbidity (i.e., at least two chronic conditions). In SNAC-K, the median age was 72.4 (IQR = 66–84; range 60–104), and 64.9% of participants were female. In total, 9.8% of the participants were affected by disability (at least one limitation in ADLs) and 87.2% was affected by multimorbidity. The median follow-up time in the HSD and SNAC-K was 6.9 and 10.6 years, respectively. Three-year mortality risk was as high as 6.1% in the whole HSD, and 14.2% in SNAC-K. Among individuals younger than 70 years, 3-year mortality was lower than 1% both in the HSD and in SNAC-K.

**Table 1:** Baseline characteristics of the HSD and SNAC-K study populations.

	HSD cohort			SNAC-K cohort		
	Whole sample N = 308,280	60–70 years N = 148,975	> 70 years N = 159,305	Whole sample N = 3363	60–70 years N = 1304	> 70 years N = 2059
Age; median [IQR]	71.0 [65.0, 78.0]	65.0 [62.0, 67.0]	78.0 [74.0, 83.0]	72.4 [66.1, 84.2]	60.8 [60.4, 66.2]	81.2 [78.1, 87.6]
Female sex; n (%)	170,706 (55.4)	76,370 (51.3)	94,336 (59.2)	2182 (64.9)	735 (56.4)	1447 (70.3)
Geographic area						
Northern Italy; n (%)	137,831 (44.7)	65,402 (43.9)	72,429 (45.5)	–	–	–
Central Italy; n (%)	60,755 (19.7)	28,353 (19.0)	32,402 (20.3)	–	–	–
Southern Italy; n (%)	109,694 (35.6)	55,220 (37.1)	54,474 (34.2)	–	–	–
Disability; n (%)	32,706 (10.6)	11,899 (8.0)	20,807 (13.1)	327 (9.8)	11 (0.8)	316 (15.4)
≥ 2 chronic diseases; n (%)	272,176 (88.3)	125,456 (84.2)	146,720 (92.1)	2931 (87.2)	968 (74.2)	1963 (95.3)
Hypertension; n (%)	178,881 (58.0)	74,816 (50.2)	104,065 (65.3)	2277 (67.7)	797 (61.1)	1480 (71.9)
Dyslipidemia; n (%)	91,744 (29.8)	43,880 (29.5)	47,864 (30.0)	1558 (46.3)	678 (52.0)	880 (42.7)
Diabetes; n (%)	56,387 (18.3)	23,256 (15.6)	33,131 (20.8)	296 (8.8)	91 (7.0)	205 (10.0)
Heart failure; n (%)	9376 (3.0)	1768 (1.2)	7608 (4.8)	353 (10.5)	17 (1.3)	336 (16.3)
Cognitive impairment or dementia; n (%)	22,619 (7.3)	4031 (2.7)	18,588 (11.7)	322 (9.6)	6 (0.5)	316 (15.3)
Any hospitalization in the last 6 months; n (%)	11,572 (3.8)	4491 (3.0)	7081 (4.4)	296 (8.8)	69 (5.3)	227 (11.0)
Follow-up; median [IQR]	6.9 [6.1, 7.0]	6.9 [6.5, 7.0]	6.9 [5.8, 7.0]	10.6 [5.4, 10.6]	10.6 [10.6, 10.6]	7.8 [3.4, 10.6]
Follow-up time of living participants; median [IQR]	6.9 (6.8–7.0)	6.9 (6.7–7.0)	7.0 (6.9–7.0)	10.6 (10.6–10.6)	10.6 (10.6–10.6)	10.6 (10.6–10.6)
Follow-up time of dying participants; median [IQR]	3.1 (1.3–5.2)	3.4 (1.5–5.3)	3.1 (1.3–5.2)	4.1 (2.0–6.2)	5.0 (3.3–7.2)	3.9 (1.8–6.1)
1-year mortality; n (%)	7568 (2.5)	1109 (0.7)	6459 (4.1)	153 (4.5)	2 (0.2)	151 (7.3)
3-year mortality; n (%)	18,899 (6.1)	2814 (1.9)	16,085 (10.1)	477 (14.2)	27 (2.1)	450 (21.9)
5-year mortality; n (%)	28,311 (9.2)	4411 (3.0)	23,900 (15.0)	768 (22.8)	60 (4.6)	708 (34.4)

For the variables’ definitions refer to the methods section.

*HSD* Health Search Database, *SNAC-K* Swedish National Study on Aging and Care in Kungsholmen, *IQR* inter-quartile range.

Overall, more than 3.4 million deficit combinations were tested in the HSD training dataset. When we evaluated the fitness of the FIs based on the sequential addition of deficits, we found that the average c-statistics rapidly increased until an inflection point was reached corresponding to the 25 most important deficits (Table 2). Of these, 17 were chronic conditions, and the remaining eight deficits included disability and economic vulnerability, a recent (i.e., in the previous six months) record for prescription of oxygen and low molecular weight heparin, hospitalization, nutritional problems, constipation, and presence of edema. In the HSD, the prevalence of these 25 deficits ranged between 53.2% (economic vulnerability) and 0.3% (prescription of oxygen in the previous 6 months). In the HSD, the PC-FI score ranged between 0 and 0.6, whereas in the external validation cohort it ranged between 0 and 0.4. In both datasets, the median PC-FI value was 0.04 (i.e., one deficit present) and showed a gamma distribution, with a tendency towards a normal curve in older age groups (Figure S1).

**Table 2:** Distribution of deficits included in the Primary Care Frailty index (PC-FI) in the derivation (i.e., training and testing) and validation datasets.

Deficit	Prevalence (%)		
	HSD training dataset	HSD testing dataset	SNAC-K dataset
Cognitive impairment or dementia	7.4	7.3	11.1
Severe disability	5.2	5.2	5.7
Cerebrovascular disease	7.9	8.0	7.9
Solid neoplasm	11.9	11.9	8.9
COPD, emphysema and chronic bronchitis	13.7	13.6	5.0
Ischemic heart disease	13.4	13.5	14.3
Heart failure	3.0	3.0	10.4
Chronic kidney disease	6.4	6.3	33.3
Atrial fibrillation	5.3	5.4	9.6
Parkinson’s disease and parkinsonism	2.9	2.8	1.1
Previous hip fracture	1.5	1.5	6.0
Anemia	15.6	15.6	12.0
Partial/total financial support for medical expenses	53.2	53.2	7.9
Oxygen prescription in the last 6 months	0.3	0.3	0.0
Any hospital overnight staying in the last 6 months	3.7	3.8	8.8
Chronic ulcers of the skin	1.1	1.0	0.9
Bradycardias and rhythm conduction disorders	3.6	3.6	1.6
Other neurological diseases*	1.0	1.0	1.9
Constipation	1.7	1.6	1.2
Prescription of LMWH in the last 6 months	3.5	3.6	0.2
Peripheral vascular diseases	2.0	2.0	1.6
Nutritional problems	0.3	0.3	3.0
Diabetes	18.3	18.33	8.8
Schizophrenia and other delusional diseases	0.7	0.7	0.6
Edema	0.2	0.2	2.3

*HSD* Health Search Database, *SNAC-K* Swedish National Study on Aging and Care in Kungsholmen, *COPD* chronic obstructive pulmonary disease, *LMWH* low molecular weight heparin.

*This category includes among others: paraplegia, chorea, cerebellar degeneration, Duchenne palsy, sequelae of meningitis, unspecific neuro-myopathy, familiar palsy, ataxia, dystonia, cerebral stenosis, several congenital malformations of the nervous system.

Higher PC-FI scores were significantly associated with a higher mortality rate over 1, 3, and 5 years based on Cox regression models adjusted for age, sex and geographic area in the testing dataset and for age and sex in the external validation dataset (Table 3). The association was also significant after stratification by age (above/below 70 years). The same results were found when using all-cause hospitalization as an outcome. The c-statistics for mortality prediction of the PC-FI alone ranged between 0.69 (5-year mortality in the older subsample, testing dataset) and 0.84 (1-year mortality in the whole external validation cohort). For hospitalization, the c-statistics ranged between 0.59 (5-year hospitalization in the older subsample, testing dataset) and 0.67 (1-year hospitalization in the whole external validation dataset) (Table 4). None of the single deficits showed c-statistics higher than 0.58 and 0.52 in the prediction of mortality and all-cause hospitalization, respectively (Table S4).

**Table 3:** Association between the Primary Care Frailty Index (PC-FI) and mortality and hospitalization over one, three and five years.

	Training dataset (HSD) N = 184,968 (60.0%)			Testing dataset (HSD) N = 123,312 (40.0%)			External validation dataset (SNAC-K) N = 3363		
	1-year HR* (95%CI)	3-year HR* (95%CI)	5-year HR* (95%CI)	1-year HR* (95%CI)	3-year HR* (95%CI)	5-year HR* (95%CI)	1-year HR* (95%CI)	3-year HR* (95%CI)	5-year HR* (95%CI)
*Whole sample*									
Mortality									
Unadjusted	3.15 (3.07–3.23)	2.99 (2.94–3.04)	2.85 (2.80–2.89)	3.16 (3.06–3.26)	3.06 (2.99–3.12)	2.91 (2.86–2.97)	3.50 (3.00–4.08)	3.26 (2.98–3.57)	3.19 (2.97–3.44)
Adjusted**	2.27 (2.20–2.34)	2.11 (2.07–2.15)	1.99 (1.95–2.02)	2.27 (2.18–2.36)	2.15 (2.09–2.20)	2.03 (1.99–2.07)	2.07 (1.71–2.51)	1.98 (1.77–2.21)	1.99 (1.82–2.18)
Hospitalization									
Unadjusted	1.73 (1.70–1.76)	1.47 (1.45–1.49)	1.31 (1.29–1.32)	1.72 (1.68–1.76)	1.45 (1.43–1.48)	1.30 (1.28–1.32)	2.18 (2.01–2.37)	2.11 (1.98–2.24)	2.13 (2.01–2.25)
Adjusted**	1.65 (1.61–1.68)	1.39 (1.37–1.41)	1.25 (1.23–1.27)	1.64 (1.60–1.68)	1.39 (1.36–1.41)	1.25 (1.23–1.27)	1.84 (1.66–2.03)	1.69 (1.57–1.83)	1.68 (1.57–1.80)
*≤ 70 years old*									
Mortality									
Unadjusted	3.78 (3.50–4.09)	3.48 (3.30–3.67)	3.18 (3.04–3.33)	4.21 (3.83–4.62)	3.56 (3.33–3.80)	3.29 (3.11–3.48)	n.a. ***	3.37 (1.89–6.01)	4.48 (3.15–6.35)
Adjusted**	4.07 (3.75–4.42)	3.65 (3.45–3.85)	3.27 (3.12–3.43)	4.29 (3.89–4.73)	3.75 (3.50–4.02)	3.41 (3.22–3.62)	n.a.***	2.74 (1.48–5.09)	3.77 (2.59–5.48)
Hospitalization									
Unadjusted	1.57 (1.51–1.63)	1.32 (1.29–1.36)	1.16 (1.14–1.19)	1.53 (1.46–1.60)	1.31 (1.27–1.35)	1.16 (1.13–1.2)	2.93 (2.21–3.89)	2.57 (2.07–3.20)	2.56 (2.12–3.10)
Adjusted**	1.65 (1.59–1.72)	1.38 (1.35–1.42)	1.21 (1.19–1.24)	1.60 (1.53–1.68)	1.36 (1.32–1.41)	1.21 (1.18–1.24)	2.84 (2.12–3.81	2.43 (1.94–3.05)	2.39 (1.97–2.90)
*> 70 years old*									
Mortality									
Unadjusted	2.65 (2.57–2.73)	2.47 (2.42–2.52)	2.32 (2.28–2.37)	2.60 (2.51–2.71)	2.52 (2.46–2.58)	2.37 (2.32–2.43)	2.83 (2.39–3.35)	2.64 (2.39–2.92)	2.52 (2.32–2.74)
Adjusted**	2.16 (2.09–2.23)	2.02 (1.97–2.06)	1.91 (1.87–1.95)	2.13 (2.05–2.22)	2.06 (2.00–2.11)	1.95 (1.90–1.99)	2.08 (1.72–2.52)	1.96 (1.75–2.19)	1.93 (1.76–2.12)
Hospitalization									
Unadjusted	1.73 (1.69–1.77)	1.47 (1.45–1.5)	1.33 (1.31–1.35)	1.75 (1.70–1.80)	1.47 (1.44–1.5)	1.34 (1.31–1.36)	1.93 (1.75–2.13)	1.81 (1.67–1.95)	1.79 (1.67–1.92)
Adjusted*	1.66 (1.62–1.7)	1.42 (1.39–1.44)	1.29 (1.27–1.31)	1.68 (1.63–1.73)	1.42 (1.39–1.45)	1.30 (1.27–1.32)	1.75 (1.57–1.95)	1.63 (1.50–1.77)	1.62 (1.50–1.75)

*HSD* Health Search Database, *SNAC-K* Swedish National Study on Aging and Care in Kungsholmen, *n.a.* not available.

*HR are reported for 0.1 increase in the PC-FI.

**Age, sex and geographic area (Northern, Central, and Southern Italy) adjustment for the training and internal validation datasets, age and sex adjustment for the external validation datasets.

***Two cases of death among younger persons in the external validation dataset—model not fitted.

**Table 4:** Prediction accuracy (c-statistics) of the Primary Care Frailty Index (PC-FI) for mortality and hospitalization.

	Training dataset (HSD) N = 184,968 (60.0%)			Testing dataset (HSD) N = 123,312 (40.0%)			External validation dataset (SNAC-K) N = 3363		
	1-year c-stat	3-year c-stat	5-year c-stat	1-year c-stat	3-year c-stat	5-year c-stat	1-year c-stat	3-year c-stat	5-year c-stat
*Whole sample*									
Mortality									
	0.79	0.76	0.74	0.79	0.76	0.74	0.84	0.81	0.79
Hospitalization									
	0.62	0.61	0.60	0.62	0.60	0.59	0.69	0.67	0.66
*≤ 70 years old*									
Mortality									
	0.75	0.72	0.69	0.77	0.71	0.69	n.a.*	0.66	0.71
Hospitalization									
	0.62	0.60	0.58	0.61	0.60	0.58	0.65	0.61	0.60
* > 70 years old*									
Mortality									
	0.74	0.71	0.69	0.73	0.71	0.69	0.78	0.75	0.72
Hospitalization									
	0.61	0.59	0.59	0.61	0.59	0.59	0.65	0.64	0.63

C-statistics are based on the unadjusted Cox regression models shown in Table 3.

*HSD* Health Search Database, *SNAC-K* Swedish National Study on Aging and Care in Kungsholmen, *n.a.* not available.

*Two cases among younger persons in the external validation dataset—model not fitted.

**Figure 1:**
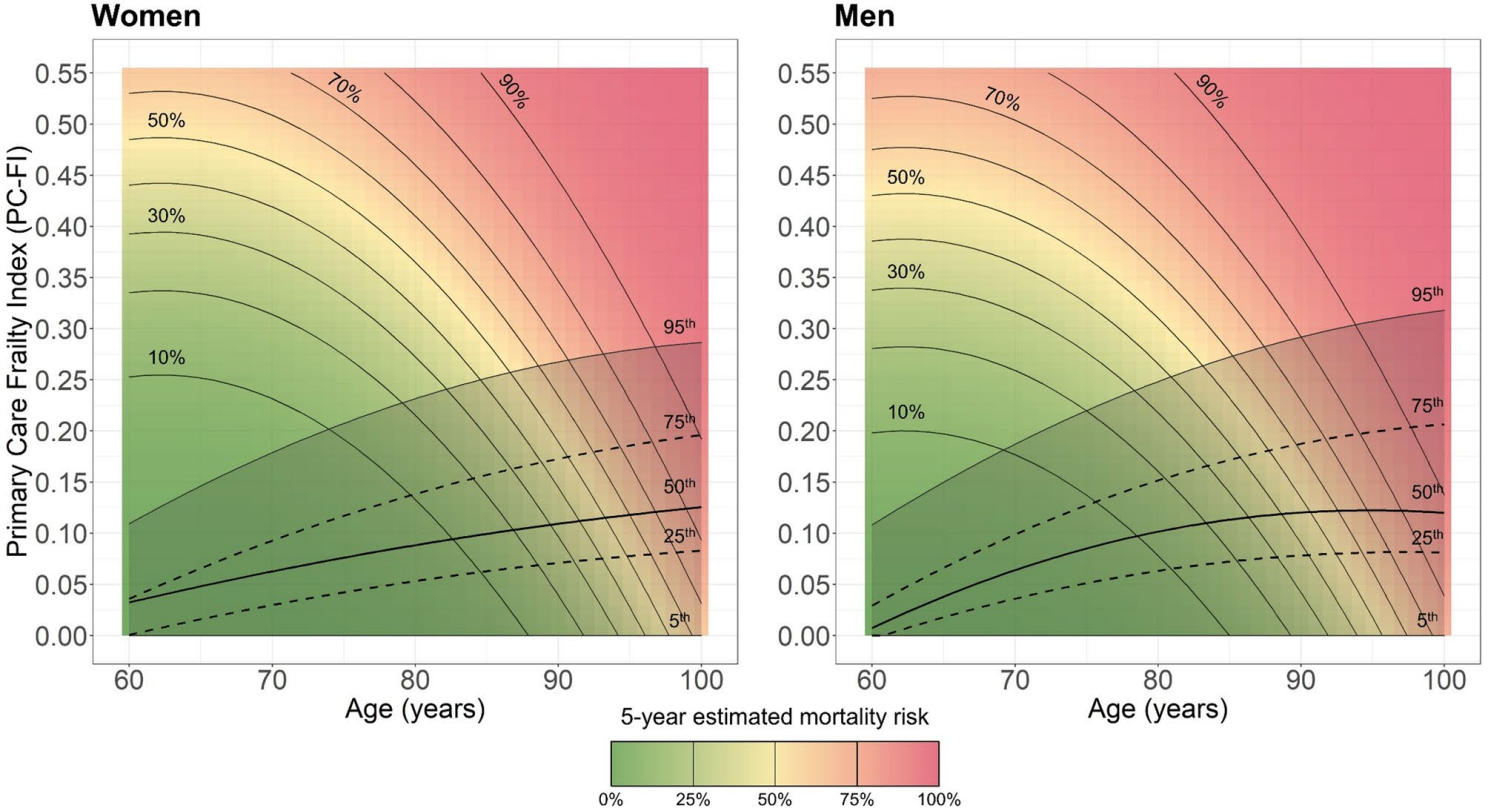
Heatmaps showing the percentile distribution and the estimated risk of death at five years of the Primary Care Frailty Index (PC-FI) by sex and age.

The age-specific percentiles of PC-FI were calculated for the HSD and plotted over two sex-specific heatmaps whose colors represent 5-year risk of death (Fig. 1).

In the HSD, 51.1% (51.6% of men; 50.7% of women) of the study population was characterized by the absence of frailty, 34.2% (33.2% of men; 35.0% of women) by mild frailty, 10.9% (11.2% of men; 10.7% of women) by moderate frailty, and 3.8% (4.0% of men; 3.6% of women) by severe frailty. Table S5 shows the distribution of frailty statuses by age and sex. As shown in Table 5, in the HSD, the incidence rate of death ranged between 8.2 events per 100 persons/year among those without frailty to 116.0 events per 1000 persons/year among those with severe frailty. Unadjusted and adjusted hazard ratios for mortality of different frailty groups in the HSD and SNAC-K are also reported. Figure S2 shows the Kaplan Meier survival curves for different frailty groups in the HSD. The psychometric properties of the different states of PC-FI are reported in Table S6 and Figure S3.

**Table 5:** Hazard ratios (HR) and 95% confidence intervals (95%CI) for mortality by frailty categories in HSD and SNAC-K, over all the available follow-up.

	HSD			SNAC-K		
	Incidence rate Deaths per 1000 persons/year	Unadjusted HR (95% CI)	Age, sex, and geographic area adjusted HR (95% CI)	Incidence rate Deaths per 1000 persons/year	Unadjusted HR (95% CI)	Age and sex adjusted HR (95% CI)
*Frailty categories*						
Fit	8.2	*Ref*	*Ref*	18.8	*Ref*	*Ref*
Mild frailty	22.2	2.7 (2.6–2.8)	1.65 (1.61–1.70)	75.9	4.0 (3.49–4.59)	1.99 (1.73–2.30)
Moderate frailty	55.5	6.7 (6.5–6.9)	2.97 (2.88–3.06)	176.0	9.25 (7.91–10.8)	3.28 (2.77–3.87)
Severe frailty	116.0	14.0 (13.5–14.5)	5.05 (4.88–5.23)	254.0	13.5 (11.0–16.7)	4.41 (3.54–5.49)

*HSD* Health Search Database, *SNAC-K* Swedish National Study on Aging and Care in Kungsholmen.

As part of the demonstration of the convergent validity of the new tool using the SNAC-K cohort, the PC-FI was found to be associated with physical frailty (odds ratio 4.25; 95%CI 3.41–5.33; area under the curve 0.84), measured as proposed by Fried et al.^19^. Moreover, the presence of moderate/severe frailty according to the PC-FI was significantly correlated with physical frailty (R = 0.429 95%CI 0.398–0.460; *p* < 0.001). In addition, the PC-FI scores was shown to be significantly associated with baseline limitations in at least one ADL or IADL, slow walking speed, low grip strength, and higher time to perform the chair stand test. Over the follow-up, PC-FI scores were associated with 3-year injurious falls, 6-year incident ADL disability, 6-year incident IADL disability, and 6-year incident dementia. The results were consistent after the adjustment for age and sex. The area under the curve of the PC-FI score in the prediction of cognitive and functional outcomes in the external validation cohort ranged between 0.61 and 0.77 (Table 6).

**Table 6:** Association of the Primary Care Frailty Index (PC-FI) with baseline functional measures and incident clinical conditions in the external validation dataset (SNAC-K).

	Unadjusted OR (95% CI)	Age and sex adjusted OR (95% CI)	AUC from unadjusted models
*Baseline functional measures*			
≥ 1 ADL limitation	4.89 (4.16–5.70)	2.75 (2.30–3.31)	0.85
≥ 1 IADL limitation	6.27 (5.39–7.34)	3.29 (2.77–3.92)	0.83
Walking speed < 0.8 m/s	6.81 (5.86–7.96)	3.16 (2.67–3.76)	0.81
Low grip strength	2.78 (2.36–3.29)	1.51 (1.25–1.83)	0.66
Physical frailty (Frailty Phenotype)	8.28 (6.79–10.20)	4.25 (3.41–5.33)	0.84
Chair stand test > 17 s	4.55 (3.97–5.23)	2.10 (1.81–2.45)	0.75
*Incident clinical conditions*			
3-year injurious falls	2.04 (1.77–2.34)	1.30 (1.09–1.54)	0.68
≥ 1 ADL limitation, 6 years*	4.02 (3.09–5.25)	2.12 (1.56–2.87)	0.77
≥ 1 IADL limitation, 6 years**	4.64 (3.59–6.03)	2.36 (1.76–3.17)	0.73
Incident dementia, 6 years***	1.85 (1.41–2.41)	1.62 (1.22–2.14)	0.61

*SNAC-K* Swedish National Study on Aging and Care in Kungsholmen, *AUC* area under the curve, *ADL* activities of daily living, *IADL* instrumental activities of daily living.

*Persons with at least 1 ADL limitation at baseline were excluded; **persons with at least 1 IADL limitation at baseline were excluded; ***persons with a dementia diagnosis at baseline were excluded.

In HSD the PC-FI and the eFI showed a correlation of 0.783 (*p* < 0.001). The eFI resulted significantly and independently associated with mortality (c-statistics of 0.671) and hospitalization (c-statistics 0.582).

## Discussion

In this study, by using electronic primary healthcare records from more than 300,000 individuals, we developed and validated a frailty index that can be generated automatically to support GPs in their clinical decision making and may instantaneously provide information on the frailty/health status of single individuals or groups of interest. We found that 51.1% of Italian primary care patients ≥ 60 years old were classified as non-frail, 34.2% presented with mild frailty, 10.9% with moderate frailty, and 3.8% with severe frailty, with women being frailer than men. The PC-FI predicted mortality and hospitalization independently of age, sex and geographical area. In an external dataset, higher levels of PC-FI were associated with physical frailty and were shown to independently predict mortality and hospitalization, as well as several other outcomes related to frailty itself, including disability, poor physical performance, injurious falls, and dementia.

Frailty poses important challenges for older patients, clinicians, and the healthcare system, threatening the sustainability of the latter. There is large agreement in the international scientific community on the need to recognize and quantify frailty in the population, and the urgency to find reliable and relatively inexpensive tools to be used on a large scale^9,15,21,22^. Of the several frailty scales proposed so far, the FI is one of the most successful ones, being based on the appraisal of individual health deficits established a priori. Despite its simplicity, the FI has consistently shown a high predictive power across several health-related outcomes^9^. The possibility to use routinely collected healthcare data to build FIs, which avoids overburdening the healthcare professional, makes this tool even more promising^17^. There are few examples of FIs developed based on primary care electronic health records, and they mainly replicate the UK eFI proposed by Clegg et al. The eFI showed similar results as the original one in the primary care settings of Australia and Canada^31,32^. The UK e-FI was built in a population of ≥ 65 years old-primary care patients, it includes 36 deficits and was independently associated with mortality, emergency admissions and institutionalization. In a follow-up study the UK eFI also showed a convergent validity with other frailty measures^18,33,34^. The authors showed a distribution of frailty in the UK population similar to what we found in the present study^18^. Overall, the e-FI reported a lower discriminative ability compared with our tool in the prediction of mortality and hospitalization. The UK eFI was built based on health deficits selected upon clinical consensus. Our previous findings suggest that a data-driven deficit selection could improve the predictivity of a FI^23^. Moreover, the UK eFI includes deficits that are assumed to be permanent. While this may be the case for several chronic diseases (e.g., heart failure, diabetes), the same might not apply to some signs and symptoms, which might only reflect transient acute episodes (e.g., dyspnea, dizziness). Limiting the inclusion to presumably chronic deficits may lead to the potential overestimation of frailty status for patients recovering from specific conditions, and to the impossibility to observe improvements in the frailty status because deficits can only “pile up” over time.

Our PC-FI introduces several elements of novelty compared with previously proposed FIs. First, the PC-FI was derived following a data-driven approach based on an optimization algorithm that allows us to get the most out of the available information^23^. While primary care health records have the advantage to cover the entire population, collected information does not always come with sufficient clinical detail. For this reason, an accurate choice of the deficits to be included in a FI is key to build a reliable and powerful predictive tool, even in the face of under-reported information. Differently from previously proposed FIs that were based on deficits selected a priori from existing lists or built following researchers’ clinical judgment, we identified 25 deficits based on their combined optimal discriminative ability in mortality prediction, not only in the whole sample but also within age, sex, and geographical subgroups. The employment of a *genetic algorithm* helped us to properly handle variables with missing or under-reported data and/or other unknown biases that may arise when data are collected following clinical rather than research purposes. Indeed, the *genetic algorithm* is blind to the pathophysiological links between deficits, frailty, and mortality and selects frailty indices by merely evaluating their performance in the training dataset. Second, for the first time, we included transient health deficits in a FI. Frailty is commonly understood as a chronic condition, and the deficits included in FIs are therefore most often permanent health problems. In our PC-FI, five deficits contribute only transiently to the score, namely if any of the following were detected in the last six months prior to the assessment: prescription of low molecular weight heparin, oxygen prescription, any hospitalization, stypsis/constipation, and edema. This allows for the PC-FI to fluctuate over time, better capturing changes in the clinical status of a patient. Third, we validated the PC-FI externally by using data from a Swedish population-based study where extensive clinical and functional information has been comprehensively assessed across participants. We have shown that, beyond mortality and hospitalization, the PC-FI is associated with several other negative outcomes, among which is physical frailty, operationalized as proposed by Fried et al.^19^. Most FIs previously proposed in the literature have been built following the recommendations initially provided by Mitnitski et al.^20^, but many lack a proper internal and external validation against strong outcomes and different operationalizations of frailty. Moreover, differently from our PC-FI, their predictive power was rarely shown to be stable across different age and sex groups. Fourth, taking advantage of a large sample representative of the older Italian primary care population, we provide sex-specific frailty charts showing the distribution (i.e., percentiles) of the PC-FI score in the reference population by age. Such nomograms, similarly to the growth charts available for the pediatric population, may allow comparing the frailty status of one same patient over time or of different patients of the same age.

The timely identification of older persons affected by frailty in the community may contribute to improved care delivery from several perspectives. GPs may be able to easily target those patients that are clinically unstable and at a higher risk of mortality, hospitalization, injurious falls, and cognitive decline. This could have direct implications in, for example, the prioritization of those patients that would benefit most from initiatives such as specific vaccination campaigns or awareness efforts during a seasonal heat wave. It may also benefit those patients that will soon need extra medical or institutional care by prompting a discussion with physicians and their caregivers about preferences in late life. At the same time, the identification of patients with severe frailty may help ensure that services are oriented towards those outcomes that are most relevant to older people, avoiding unnecessary interventions^35^. All of this could reduce the number of avoidable and inappropriate hospitalizations and emergency room visits for these patients. Notably, the availability of continued frailty assessments by GPs (i.e., in stable health conditions) would also be helpful during acute episodes of care, when a proper baseline frailty assessment is likely challenged by the ongoing emergent problem. Moreover, the early identification of primary care patients with mild frailty may trigger interventions aimed at preventing the further development of more severe frailty, as already shown in recent intervention studies^36,37^. The sex-specific frailty charts provided herein may enable the detection of unexpected deviations from a patient’s reference percentile, promoting further clinical workup. From a public health standpoint, the stratification of the population by frailty levels would allow acquiring a bird’s-eye view of the frailty case-mix in each practice, town, or region. This would facilitate, among others, a more rational and equitable allocation of both economic and human resources. Moreover, the country-level implementation of the PC-FI would eventually provide regional and central governments with the possibility to monitor population-level health trends and appraise the effectiveness of different health policies.

The results of our study should be read considering some limitations. First, electronic health records, including primary care health records, may suffer from a certain level of information under-reporting, which is especially true for what concerns signs and symptoms. Differential misclassification may also arise in this kind of datasets. At the same time, patients displaying a more intense utilization of healthcare systems are more likely to receive new diagnoses. Moreover, to be a reliable source of information, electronic health records require to be regularly updated. For this reason, in our study we included only primary care patients with at least 5 year-records with the same GP. This might affect the generalizability of the PC-FI to individuals with shorter historical data. However, when comparing this subgroup with the whole population of individuals 60+, both registered in the HSD and in the general Italian population—according to national statistics—they present with identical demographic characteristics. Furthermore, the high predictive performances shown by our tool in the external validation population is reassuring in terms of generalizability. Moreover, the genetic algorithm maximizes the utilization of all available data by tailoring the FI to the outcome of interest, even in the face of a potential information bias. Second, the PC-FI has not been validated in patients under 60 years that represent a significant proportion of primary care patients, and who can present with complex clinical profiles triggered by single diseases, as for example multiple sclerosis or other autoimmune diseases. However, the activation of specific diagnostic and therapeutic pathways is usually performing well in younger populations, where major burdensome diseases are promptly identified and managed according to clinical guidelines. Third, the *genetic algorithm* is prone to overfit the deficit selection on the outcome considered, which was mortality rate, in our case. However, several statistical specifications to the algorithm and the stratification of the analyses by age, sex and geographical area have been used precisely to avoid overfitting. The results obtained through the internal and external validation, including several other outcomes beyond mortality, confirm the flexibility of our methodology. Fourth, a higher richness of information of socioeconomic status of primary care patients would have probably ended up in the selection by the genetic algorithm of these variables, as arguably poor education. However, we have been able to capture—at least partially—this domain by identifying those primary care patients with a financial exemption for medical expenses due to financial disadvantages. Fifth, as we have reported in the present study, same PC-FI frailty categories might identify individuals with different mortality rates across different settings (i.e., a Swedish population-based study in our case). The distribution of sociodemographic and clinical characteristics might be responsible for such discrepancies, which disappeared in adjusted survival analyses. Therefore, caution should be adopted in applying our index outside a primary care population. Finally, death information in the HSD might suffer from some under-reporting given that it is not derived from registries but based rather on the recording by the GPs. Still, validation studies have shown the reliability of such information^38^.

In conclusion, we were able to identify and quantify frail primary care patients by developing and validating a trustable FI. The PC-FI was built on a large representative sample of more than 300,000 Italian primary care patients ≥ 60 years old and validated both internally and in a well-characterized population-based cohort of older Swedish adults. The implementation of an automated computation of the PC-FI in the clinical software routinely used by GPs would assist them in their clinical decision making by guiding prioritization at the individual and collective levels. Finally, the availability of such a measure on a large scale may be used to monitor health and frailty at the population level across geographical areas and over time.

## Supplementary Information

Supplementary Information.

## Data Availability

The datasets generated and analyzed during the current study are not publicly available due to proprietary seasons, but are available from the corresponding author on reasonable request.
