# Supplementary material for: Frailty detection among primary care older patients through the Primary Care Frailty Index (PC-FI)
Source: Sci Rep. 2023 Mar 2;13:3543. doi: 10.1038/s41598-023-30350-3 (PMC9981758; doi:10.1038/s41598-023-30350-3)
Supplement: Supplementary file 1 — Supplementary Information. [file 41598_2023_30350_MOESM1_ESM.docx]

**Supplementary methods**

The genetic algorithm (GA) is a recursive algorithm. In the first iteration, an initial set of Frailty Indices (FI) (or “solutions”, in GA terminology) is created. This set of FIs is also called “initial population”. These solutions represent the starting point used by the *genetic algorithm* to search (and create) more fit solutions to the problem. Our *genetic algorithm* created 1500 FIs. Previous studies reported the initial number of solutions to be at least 10 times the number of potential variables to be included. After different trials, we found that this number offered a good balance between late convergence and time of execution. Each potential deficit had a uniform probability to be included (or excluded) from each FI. Each randomly created FI was represented by a vector (*chromosome* in *genetic algorithm* terminology) with dimension 101 (total number of potential deficits in HSD): a 1 or 0 (*allele* in *genetic algorithm* terminology) was recorded for each vector component, according to the status of the deficit (included or excluded, respectively). In this phase, the number of deficits included (i.e. the number of “1”s recorded) in each FI ranged between 15 and 70. To note, we did not introduce any constraints on the number of deficits to be considered in the next phases of the algorithm. A FI was built from each vector; the sum of the deficits included was divided by the total number of deficits taken into consideration.

Each potential FI is then evaluated and its “fitness” is calculated. The *genetic algorithm* assesses the *fitness* of each solution according to the definition of *goodness* set by the developing team. Our *genetic algorithm* was coded to find the FI with the highest discriminative ability for the prediction of mortality in the whole study population and different subsamples. The discriminative ability was assessed calculating the mathematical mean between the concordance statistic in the prediction of mortality during the whole follow-up period, exhibited in the whole study sample and in seven subsamples (i.e. males and females, individuals younger and older than 70 years old, participants living in Northern, Central, and Southern Italy). The C-statistics were obtained from unadjusted Cox regression models run in a random subsample of 10,000 participants drawn from the training subsample of HSD. The random subsample was re-drawn in each run of the genetic algorithm.


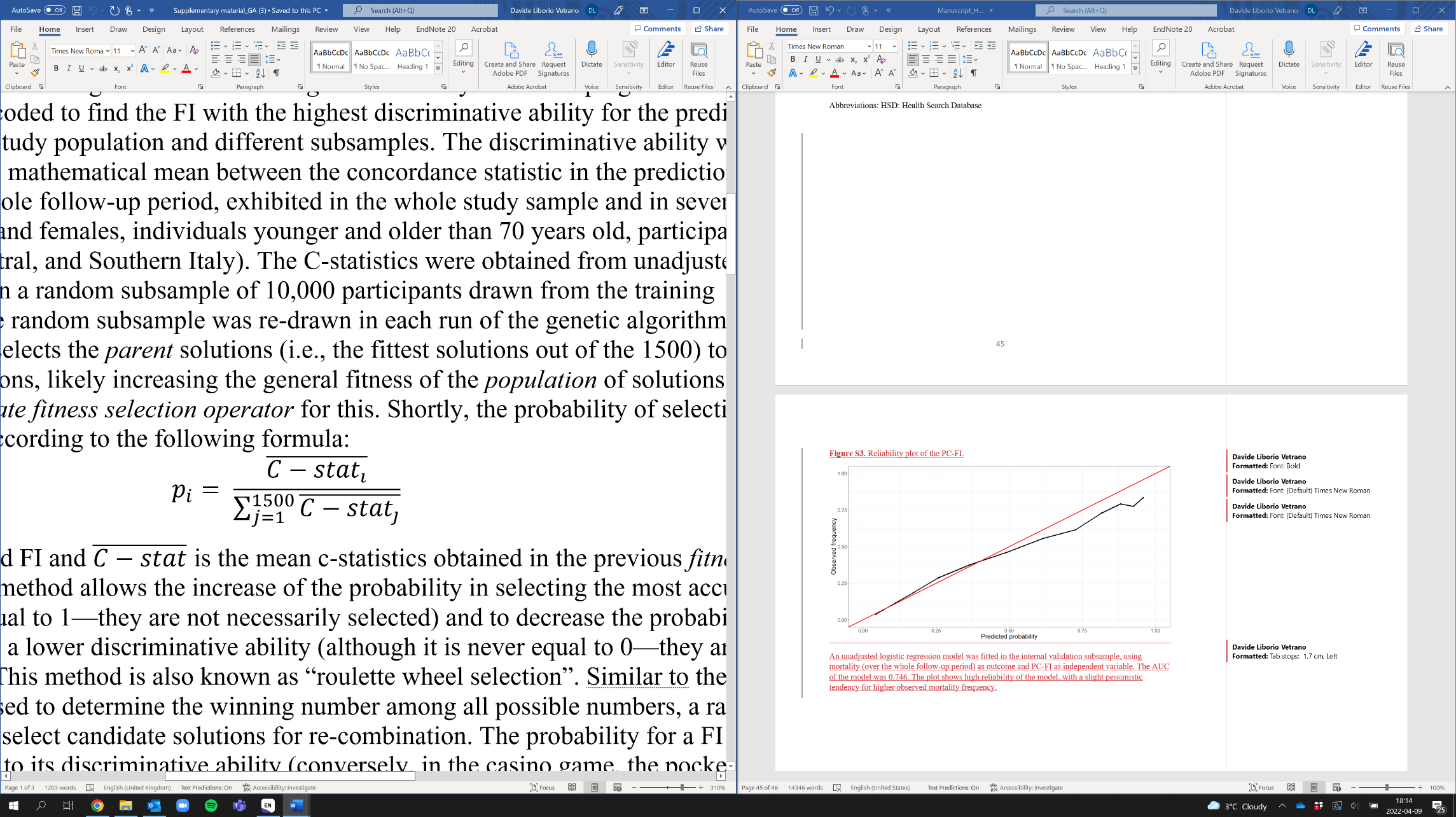
The *genetic algorithm* selects the *parent* solutions (i.e., the fittest solutions out of the 1500) to be re-combined in new solutions, likely increasing the general fitness of the *population* of solutions. We employed a *proportionate fitness selection operator* for this. Shortly, the probability of selection for each FI was obtained according to the following formula:

where *i* is the considered FI and *C – stat* is the mean c-statistics obtained in the previous *fitness evaluation* phase. This method allows the increase of the probability in selecting the most accurate FIs (although it is never equal to 1—they are not necessarily selected) and to decrease the probability in selecting those FIs with a lower discriminative ability (although it is never equal to 0—they are not necessarily excluded). This method is also known as “roulette wheel selection”. Similar to the casino game, where a ball is used to determine the winning number among all possible numbers, a random probability *p_i_* is used to select candidate solutions for re-combination. The probability for a FI to be selected is proportional to its discriminative ability (conversely, in the casino game, the pocket in the roulette wheel in which the ball may stop is equally probable for all numbers). We obtained 1470 couples of FIs for the *re-combination* phase: the 30 FIs with the highest c-statistic in every iteration were maintained for the subsequent iteration (*elitism* = 2%). There were no constraints on the number of times a FI could be selected and the same FI could also be selected for both elements of the couple (therefore, the recombination phase would not have any effect).

In the next phase, the *genetic algorithm* re-combines the selected solutions (*parents*) to generate *children* solutions (*offspring* in *genetic algorithm* terminology). Each *child* solution includes sections of the *parents’* *chromosome*. In our study, two random numbers were generated for each couple of selected FIs. These numbers, called *crossing-over points* in *genetic algorithm* terminology, identified the “switching” positions in the vectors representing the FIs. A new vector (i.e. a new FI) was created by combining the components comprised of the first component and the component identified by the first random number of the first vector, the components comprised of the positions identified by the first and the second random numbers of the second vector, and the components comprised of the second random number and the last component of the first vector again. This procedure was repeated for all the couples selected. To note, the *genetic algorithm* has a low but existing probability to randomly change part of a *chromosome*. This phase allows the *genetic algorithm* to find new solutions that in theory cannot be generated starting from the *initial population*. On the other side, high *mutation* *rates* can possibly change solutions with high *fitness*. In our *genetic algorithm*, we established a 7.5% chance that a random component of a vector was changed (therefore excluding or including random deficits).

In the last phase of each iteration, the *genetic algorithm* replaces the solutions included in the *initial population* with those generated in the *re-combination phase*. In our *genetic algorithm*, re-combined FIs replaced the 98% least accurate FIs in the *initial population*. The *genetic algorithm* ran until at least one of the following conditions was met: 1) 100 iterations were reached, or 2) the average C-statistics of the best FI did not show any increase for four consecutive iterations.

We run the *genetic algorithm* 50 times, therefore starting from 50 different *initial populations*, and examining 50 random samples of 10,000 HSD participants drawn from the training subsample. We counted the number of times each deficit was included in the FI with the highest c-statistic in each iteration. In total, the GA performed 2224 iterations. All 101 deficits were sorted according to the number of times they were selected, out of the total number of iterations. We then proceeded to create 109 FIs, including an increasing number of deficits, and evaluating their c-statistics on the whole train dataset for the prediction of mortality (whole follow-up time) in the whole sample and in the 7 subsamples previously stated (i.e., sex, age, and geographical area). The first FI created only included the most selected deficit, the second FI included the two most selected deficits, whereas the 109^th^ FI created included all deficits. As already shown in a previous paper (10.18632/aging.103118), the average c-statistics increased with an increasing number of deficits, then exhibited a plateau and a subsequent decreasing pattern**1**. The HS-FI was the results of the 25 most selected deficits, whose combination achieved the highest c-statistic according to the methodology previously described.

**Table S1.** Participants’ characteristics and outcomes distribution in the HSD testing and training datasets.

|  | Testing dataset  N = 123,312 (40.0%) | Training dataset  N = 184,968 (60.0%) | *p* |
| --- | --- | --- | --- |
| Age; median [IQR]) | 71.00 [65.00, 78.00] | 71.00 [65.00, 78.00] | 0.610 |
| Female sex; n (%) | 68150 (55.3) | 102556 (55.4) | 0.329 |
| Geographic area |  |  | 0.139 |
| Northern Italy; n (%) | 55385 (44.9) | 82446 (44.6) | |
| Central Italy; n (%) | 24278 (19.7) | 36477 (19.7) | |
| Southern Italy; n (%) | 43649 (35.4) | 66045 (35.7) | |
| Disability | 12951 (10.5) | 19755 (10.7) | 0.118 |
| ≥2 chronic diseases; n (%) | 108866 (88.3) | 163310 (88.3) | 0.964 |
| Hypertension; n (%) | 71534 (58.0) | 107347 (58.0) | 0.894 |
| Dyslipidemia; n (%) | 36919 (29.9) | 54825 (29.6) | 0.076 |
| Diabetes; n (%) | 22588 (18.3) | 33799 (18.3) | 0.756 |
| Heart failure; n (%) | 3750 (3.0) | 5626 (3.0) | 1.000 |
| Cognitive impairment or dementia; n (%) | 9005 (7.3) | 13614 (7.4) | 0.553 |
| Any hospitalization in the last 6 months; n (%) | 4702 (3.8) | 6870 (3.7) | 0.160 |
| Any contact with specialist in the last 6 months; n (%) | 70564 (57.2) | 105809 (57.2) | 0.915 |
| Follow-up (median [IQR]) | 6.93 [6.09, 6.98] | 6.93 [6.09, 6.98] | 0.857 |
| 1-year mortality; n (%) | 3058 (2.5) | 4510 (2.4) | 0.472 |
| 3-year mortality; n (%) | 7642 (6.2) | 11257 (6.1) | 0.209 |
| 5-year mortality; n (%) | 11387 (9.2) | 16924 (9.1) | 0.429 |

Abbreviations: HSD: Health Search Database; IQR; interquartile range

**Table S2:** List of the101 deficits considered for the development of Primary Care Frailty Index (PC-FI) in the HSD.

| Deficit | Category | Coding | ICD-9 codes | Exemptions | ATC codes | National codes |
| --- | --- | --- | --- | --- | --- | --- |
| Pneumonia or bronchitis in the last 6 months | acute condition | 0,1 [0,1] | 507, 480, 481, 482, 483, 485, 486, 466 |  |  |  |
| Other acute infective condition in the last 6 months | acute condition | 0,1 [0,1] | 507, 480, 481, 482, 483, 485, 486, 466, 599, 780.6, 788.1, 788.63 |  |  |  |
| Any new prescription of antibiotics in the last 6 months | acute condition | 0,1 [0,1] |  |  | J01 , J02 , J05 |  |
| Any new low-molecular weight heparin prescription in the last 6 months | acute condition | 0,1 [0,1] |  |  | B01AB |  |
| Any new oxygen prescription in the last 6 months | acute condition | 0,1 [0,1] |  |  | V03AN01 |  |
| Urinary tract infection in the last 6 months | acute condition | 0,1 [0,1] | 599, 788.1, 788.63, 788.1, 788.63 |  |  |  |
| Allergic conditions | chronic disease | 0,1 [0,1] | 477,493.0 , 558.3, 691.8 , 692.3 , 692.4, 692.5, 692.6 , 692. 9, 708 , V07.1, 690.12 , 692.81 , 692.84, 692.89 |  |  |  |
| Anaemia | chronic disease | 0,1 [0,1] | 280, 281, 282, 283, 285, 284.01,284.09, 284.81, 284.89 | M003 | B03A , B03XA |  |
| Asthma | chronic disease | 0,1 [0,1] | 493 | M007 | R03DC , R03BC |  |
| Atrial Fibrillation | chronic disease | 0,1 [0,1] | 427.3 |  |  |  |
| Other autoimmune diseases | chronic disease | 0,1 [0,1] | 443.1, 694.4, 694.5, 696, 710, 725, 446, 701.0, 517.2, 135 | M028 , M030 , M045 , M054 , M067, M059 | D05 |  |
| Blindness and visual loss | chronic disease | 0,1 [0,1] | 369, V52.2,V43.0 | C05 |  |  |
| Blood or blood-forming organs diseases | chronic disease | 0,1 [0,1] | 286, 287, 279, 288.4, 288.1, 288.2, 289.4, 289.6, 289.83, 289.9, 289.51 |  |  |  |
| Bradycardias or other cardiac conduction abnormalities | chronic disease | 0,1 [0,1] | 426, 427.81, V45.01, 746.86 |  |  | 89.48.1, NKIPT11, , 37.87, PACC118, PACC551, PACC552, 89.50.2 |
| Heart valves diseases | chronic disease | 0,1 [0,1] | 394,395,397,396,424.0,424.1,424.2,424.3,V43.3,V42.2,746.3,746.4,746.5,746.6, 746.81 |  |  |  |
| Cataract and other lens diseases | chronic disease | 0,1 [0,1] | 366.0,366.1,366.3,366.4,366.5,366.8,366.9,379.3, 366.22 |  |  |  |
| Cerebrovascular diseases | chronic disease | 0,1 [0,1] | 430, 431,432, 433, 434 , 435, 436, 437, 438, 443.21 |  |  |  |
| Congenital abnormalities | chronic disease | 0,1 [0,1] | 758 | M051 , M062 , M064 , M065 , M066 |  |  |
| Chronic infectious diseases | chronic disease | 0,1 [0,1] | 011.0 , 011.1, 011.2, 011.3 , 011.4, 011.5, 011.7, 011.8, 011.9, 010, 012.0 , 012.1 , 012.2, 012.3, 012.8, 013.1, 013.2, 013.4, 013.8, 013.9, 014.8, 015, 016, 017.0, 017.2, 017.3, 017.4, 017.5, 017.6, 017.7, 017.8, 017.9, 018.8, 018.9, 030, 031, 090, 092, 093, 094, 095, 096, 097, 103, 046, 042, 086, 120, 139, 730.1, 104.0, 102.4, 102.5, 102.6, 102.7, 102.8, 102.9, 088.81, 114.4, 115.05, 116.0, 137.1, 137.2, 137.3, 137.4 | M016 , M020 , M055 |  |  |
| Chronic kidney disease | chronic disease | 0,1 [0,1] | 582, 585, 587, 588, 589, 753.1, 403.91, 404.92, 404.93, 753.0, V45.73, V42.0 | M023 , M061 , M062 |  | 38.95, 39.95.1, , 39.95.4, 39.95.9, 54.98.1, 54.98.2, 88.49.1, 54.93, 90.93.3, 90.30.4, 54.98.7, 54.98.A, 38.95.9, 38.95.A, 39.43, 39.95.C, 39.95.D, 39.95.E, 39.95.F, 39.95.G, 39.95.H, 39.95.I, 39.95.O, 39.95.P, 39.95.Q, 39.99.3, 89.03.1, 90.16.3, 90.24.3, 90.37.4, 90.40.4, 90.43.5, 90.44.1, 39.95.A, 54.98.4, 89.03, 89.07, 93.01.C, 93.01.D, 39.95.B, 54.98.5, 5493, PAC25, PAC26, 54983, 39955, 39.4, 39.27.1, 39.96.1, 39.96.2, 39.42, 39.27 |
| Chronic liver disease | chronic disease | 0,1 [0,1] | 070.3, 070.7, 571, 070.42, 070.44, 070.54, 572.3, 572.8, 573.0, 573.4, 573.5, 751.62, V42.7 | M008 , M016 |  |  |
| Chronic pancreatic/gallbladder disease | chronic disease | 0,1 [0,1] | 574.1, 574.2, 574.4, 574.5, 574.7, 574.8, 574.9, 577.1, 577.8, 575.11, 575.12, 575.2, 575.5, 575.6, 575.9, 576.0, 576.4, 576.9, 751.61, 751.69, 751.7, 782.4 | M042 | A09AA02 |  |
| Chronic ulcers of the skin | chronic disease | 0,1 [0,1] | 707, 454.0,454.2 |  |  |  |
| Colitis and intestinal conditions | chronic disease | 0,1 [0,1] | 558.1, 564.1, 564.5, 564.7, 564.8, 455, 557.1, 557.9, 562.00, 562.02, 562.10, 562.12 |  |  |  |
| COPD/emphysema | chronic disease | 0,1 [0,1] | 491, 492, 494, 496 | M057, M024 | R03BB |  |
| Deafness and other chronic hearing problems | chronic disease | 0,1 [0,1] | 387, 389, 744.0, V53.09, V53.2, V43.89, V41.2 | C06 |  | 2145120060, 2145001090, 2145001120, 2145001150, 2145001180, 2145001210, 2145001240, 2145060030, 2145060060, 2145060090, 2145090030, 2145090060, 2145120030 |
| Dementia or cognitive decline | chronic disease | 0,1 [0,1] | 290, 294.1, 294.2, 331.1, 331.2, 331.0, 331.6, 331.7, 331.8, 331.9, 330, 797, 780.83 | M011 , M029 , M049 , C02 | N06DA , N06DX01 |  |
| Depression and other mood disorders | chronic disease | 0,1 [0,1] | 296, 298.0, 300.4 |  |  |  |
| Diabetes | chronic disease | 0,1 [0,1] | 250, 251.3 | M013 | A10 |  |
| Dorsopathies | chronic disease | 0,1 [0,1] | 737.1,737.2, 732, 724.0, 724.1, 720, 722.9, 724.8, 724.9, 756.1, 754.2, 756.16 |  |  |  |
| Dyslipidaemia | chronic disease | 0,1 [0,1] | 272 | M025 |  |  |
| ENT chronic conditions | chronic disease | 0,1 [0,1] | 380.2, 382.1, 382.2, 382.3, 385.3, 384.1, 385.1, 386, 383.3, 998.1, 998.2, 477.9, 472, 473, 471, 470, 478, 474, 476, 478.3, 523.1, 749, 750.0, 750.1, 750.2, 997.99, 478.19, 478.74, 527.0, 527.6, 527.7 |  |  |  |
| Epilepsy | chronic disease | 0,1 [0,1] | 345 |  |  |  |
| Esophagus/stomach/duodenus conditions | chronic disease | 0,1 [0,1] | 456.0, 456.1, 456.2, 530.2, 530.3, 530.4, 530.5, 530.6, 530.7, 531.4, 531.5, 531.6, 531.7, 532.4, 532.5, 532.6, 532.7, 533.4, 533.5, 533.6, 533.7, 534.4, 534.5, 534.6, 534.7, 535.1, 535.5, 536.0, 536.3, 537.2, 537.6, 750.4, 750.8, 750.9, 530.0, 530.11, 530.81, 530.84, 530.85, 530.87, 537.83, 537.82, V45.75 |  | A02BX |  |
| Glaucoma | chronic disease | 0,1 [0,1] | 365 | M019 |  |  |
| Heart failure | chronic disease | 0,1 [0,1] | 416.2, 416.9, 425.1, 425.5, 425.7, 425.8, 425.9, 428, 402.01, 402.11, 402.91, 404.01, 404.03, 404.11, 404.13, 404.91, 404.93, 429.5, 429.6, 429.71, V42.1, V53.32, 428.21, 428.23, 428.31, 428.33, 428.41, 428.43 | M021 |  | MW_3551, MW96, MW9O, MW_4020, MW_4244, MW_4251, MW_4476 |
| Haematological conditions | chronic disease | 0,1 [0,1] | 201, 202, 203, 204, 205, 206, 207, 208, 200 |  |  |  |
| Hypertension | chronic disease | 0,1 [0,1] | 401, 402, 403, 404, 405 | MA31 , M031 |  |  |
| Inflammatory arthropathies | chronic disease | 0,1 [0,1] | 714, 274, 712, 719, 716.5, 716.6, 716.8, 713, 720, 99.3, 713.1, 714.3 | A006 | M01CB |  |
| Inflammatory bowel disease | chronic disease | 0,1 [0,1] | 555, 556 | M009 | A07E |  |
| Ischemic heart disease | chronic disease | 0,1 [0,1] | 410, 411, 412, 413, 414, V45.81, V45.82 |  | C01DA , C01EB18 |  |
| Migraine facials pain syndrome | chronic disease | 0,1 [0,1] | 346, 339, 350, 351.1, 307.81 |  | N02C |  |
| Multiple sclerosis | chronic disease | 0,1 [0,1] | 340 | M046 |  |  |
| Neurotic/stress/somatic conditions | chronic disease | 0,1 [0,1] | 300, 308 |  |  |  |
| Obesity | chronic disease | 0,1 [0,1] | 278 |  |  |  |
| Osteoarthritis and degenerative diseases | chronic disease | 0,1 [0,1] | 715, 713.2 |  |  |  |
| Osteoporosis | chronic disease | 0,1 [0,1] | 733.0 , 733.1 |  | M05BA , M05BB , M05BX03 , M05BX53 |  |
| Previous hip fracture | chronic disease | 0,1 [0,1] | 820, 821 |  |  |  |
| other MSK conditions | chronic disease | 0,1 [0,1] | 137.3, 734, 736, 738, 717, 718.0, 718.1, 718.2, 718.3, 718.5, 718.8, 718.9, 728.5, 733.7, 726.0, 729.1, 726.2,727.2, 728.6, 732.1, 732.7, 755.4, 885, 886, 887, 895, 896, 897, V49.6, V49.7, V49.4, V42.4, V43.6, V43.7, 736.89, 736.05, 736.79, 728.82, 727.03, 727.04, 728.71, 728.79, 727.61, 726.11, 738.12, 722.83, 754.31, 755.25, 755.35, 756.83, 756.59, V52.0, V52.1 | M037 , M060 |  |  |
| other cardiovascular conditions | chronic disease | 0,1 [0,1] | 393, 398, 417.1, 423.1, 423.2, 426.7, 440, 441, 442, 458, 746, 745, 747, 443.81, 427.89, V45.00, V45.09 |  |  |  |
| other digestive conditions | chronic disease | 0,1 [0,1] | 568, 579.0, 579.1, 579.2, 564.2, 751.1, 751.2, 751.5, 787.6, V45.72, V88.1, V45.3 |  |  |  |
| other eye diseases | chronic disease | 0,1 [0,1] | 374.2, 374.3, 374.4, 375.1, 375.2, 375.4, 375.5, 375.6, 375.8, 375.9 , 376.1, 376.2, 376.3, 376.4, 376.5, 376.6, 376.8, 376.9, 372.1, 371.0, 371.4, 371.5, 371.6, 371.7, 371.8, 371.9, 364.8, 636.3, 363.5, 361.0, 362.5, 362.6, 362.7, 362.4, 362.8, 362.9, 377.4, 377.9, 378.5, 743.6, 743.0, 743.1, 743.4, 743.5, 364.10, 363.41, 362.77, 362.29, 378.87, V42.5 | M041 , M053 |  |  |
| other genitourinary conditions | chronic disease | 0,1 [0,1] | 592, 594, 595.1, 595.2, 595.3, 607.9, 614.1, 615.1, 614.7, 614.6, 753.4, 753.3, 753.5, 753.6, 753.7, 753.8, 753.9, 137.2, 595.82, 596.0, 596.1, 596.2, 596.3, 596.4, 598.00, 625.6, 788.30, 788.32, 607.84, 607.0, 627.3, 752.6, 753.29, 753.2, V45.77, V43.5, V88.0 |  |  |  |
| other metabolic diseases | chronic disease | 0,1 [0,1] | 252, 253.0, 253.1, 253.2, 253.3, 253.4, 253.5, 253.6, 253.8, 253.9, 255, 256, 257, 258, 260, 261, 270, 272.7, 277.5, 271.8, 277.4, 277.6, 277.8, 277.9, 579.4, 579.8, 579.9, 579.3, 268.2, 588.1, 588.81 | M012 , M022 , M026 , M032 |  |  |
| other neurological diseases | chronic disease | 0,1 [0,1] | 137.1, 344.1, 326, 333.4, 334, 335, 333.7, 333.6, 352, 358, 359, 331.4, 331.5, 331.3, 348.8, 336.9, 349, 740, 742.1, 742.3, 742.4, 741, 742.5, 742.8, 781.2, 781.3, 333.89, 333.90, 333.94 | M017 |  |  |
| other psychiatric diseases | chronic disease | 0,1 [0,1] | 294.0, 293.8, 294.8, 310.0, 310.1, 310.2, 310.9, 303, 304, 291, 292, 302.7, 312.3, 300.19, 317, 318, 315.3, 315.4, 299, 315.8, 315.9, 307.2 | M014 |  |  |
| other respiratory conditions | chronic disease | 0,1 [0,1] | 137.0, 500, 502, 503, 504, 505, 506, 501, 508.1, 508.2, 508.8, 508.9, 748, 278.03, 518.83, V45.76, V42.6, V43.81 | M018 |  |  |
| other skin conditions | chronic disease | 0,1 [0,1] | 694.8, 698.3, 697, 708.8, 692.82, 706.8, 757.1, 757.3, 705.81, 757.33, 757.39 | M045 |  |  |
| Parkinson disease and parkinsonism | chronic disease | 0,1 [0,1] | 332, 333.0, 333.1 | M038 | N04BA , N04BC , N04BX |  |
| peripheral neuropathy | chronic disease | 0,1 [0,1] | 138, 353.9, 353.8, 354, 355, 356, 357.7, 357.8, 721.1, 721.4, 721.91 |  |  |  |
| peripheral vascular disease | chronic disease | 0,1 [0,1] | 440.2, 440.3, 440.4, 443.2, 443.9 | M036 | B01AC23 |  |
| prostate diseases | chronic disease | 0,1 [0,1] | 600.0,600.1, 601.1, 601.8 |  | G04C |  |
| schizophrenia and other delusional disorders | chronic disease | 0,1 [0,1] | 295, 297.0, 297.1, 297.2, 297.3, 295.7, 298.9 | M044 |  |  |
| sleep disorder | chronic disease | 0,1 [0,1] | 307.42, 307.44, 307.47, 307.48, 307.49 |  |  |  |
| solid neoplasms | chronic disease | 0,1 [0,1] | 140,141,142, 143, 144, 145, 146, 147, 148, 149, 150, 151, 152, 153, 154, 155, 156, 157, 158, 159, 160, 161, 162, 163, 164, 165, 170, 171, 172, 173, 174, 175, 176, 179, 180, 181, 182, 183, 184, 185, 186, 187, 188, 189, 190, 191, 192, 193, 194, 195, 196, 197, 198, 199 | M048 |  | 92.24.4, , 89.7, 92.24.5, 92.24.6, 38.99.1, 38.99.2, 89.04, 92.24.B, 92.31.1, 92.31.4, 92.40.2, 85.99.1, 85.99.2, 85.99.4, 96.59, 92.29.M, 92.24.7, 92.24.8, 92.29.H, 92.29.J, 92.29.a, 92.29.b, 92.29.c, 92.29.d, 92.29.e, 92.30.6, 92.31.3, 92.24.A, 92.40.1, 38991, 38992, PCA84, 92401, 92402, PACC409, 92.47.8, 92.47.9, 92.29.L, 92.29.K |
| thyroid disease | chronic disease | 0,1 [0,1] | 240, 241, 242, 246, 244, 245.2 | M027 , M035 , M056 | H03AA , H03B |  |
| venous lymphatic conditions | chronic disease | 0,1 [0,1] | 448, 459, 457, 454.1, 454.8, 454.9 |  |  |  |
| multimorbidity (2+ chronic disease) [sum of chronic diseases] | chronic disease | 0,1 [0,1] |  |  |  |  |
| Immunodeficiency | chronic disease | 0,1 [0,1] |  | M052 |  |  |
| Nutritional issue | chronic disease | 0,1 [0,1] | 787.2 , 262, 263, 783.2, 783.0, 783.3, 783.7, 783.9 |  |  | 97.01.1, 44.19.3, 44.19.5, 44.19.6, 93.57.2, -, 89.7, 97.51.1, 97.02.1 |
| # of GP visits in the last 6 months | Health-care system utilization | 0,1,2+ [0,0.5,1] | [software’s internal codes] | | | |
| # of x-rays/ultrasound imaging tests in the last 6 months | Health-care system utilization | 0,1,2+ [0,0.5,1] |  |  |  | 88.72.2, 88.72.3, 88.72.4, 88.72.5, 88.73.5, , 89.44, 88.76.2, 88.71.4, 92.01.3, 92.01.4, 92.02.1, 92.02.2, 92.02.4, 92.02.5, 92.03.1, 92.03.2, 92.03.3, 92.03.5, 92.04.1, 92.05.2, 92.05.3, 92.05.4, 92.05.5, 92.05.6, 92.09.3, 92.13, 92.14.1, 92.14.2, 92.15.1, 92.15.2, 92.15.4, 92.15.5, 92.16.1, 92.18.1, 92.18.2, 92.18.4, 92.18.5, 92.19.1, 92.19.2, 92.19.3, 92.19.6, 87.03.2, 87.03.7, 87.03.9, 87.06.1, 87.09.2, 87.11.1, 87.11.2, 87.11.3, 87.12.1, 87.12.2, 87.16.1, 87.16.2, 87.16.3, 87.17.2, 87.22, 87.23, 87.24, 87.29, 87.44.2, 87.49.1, 87.61, 87.62, 87.62.1, 87.62.2, 87.62.3, 87.63, 87.64, 87.65.1, 87.65.2, 87.65.3, 87.66, 87.69.1, 87.77.1, 87.79, 87.99.1, 87.99.2, 88.03.1, 88.04, 88.19, 88.21, 88.27, 88.29.1, 88.29.2, 88.31, 88.31.1, 88.35.1, 88.37.1, 88.39.1, 88.71.1, 88.72.1, 88.73.1, 88.73.3, 88.73.4, 88.74.1, 88.74.2, 88.74.3, 88.74.4, 88.74.5, 88.75.1, 88.75.2, 88.76.1, 88.78.1, 88.79.1, 88.79.3, 88.79.4, 88.79.5, 88.79.6, 88.79.7, 88.79.8, 88.90.3, 95.14, 06.11.2, 95.13, 88.79.2, 88.78.2, 92.29.1, 88.23, 88.28, 88.22, 88.26, 87.17.1, 88.73.2, MW_4262, MW_4263, 92.14.3, 92.19.7, 88.01.6, 87.03.8, 87.03.1, 87.03.4, 87.03.5, 87.03, 87.41, 87.71, 88.01.3, 88.33.2, 88.38.1, 88.38.3, 88.38.5, 88.38.6, 88.72.6, 88.72.7, 88.75.3, 88.77.3, 88.78.3, 88.78.4, 88.78.5, 88.78, 88.79.9, 88.90.1, 88.90.2, 88.91.3, 88.91.5, 88.91.6, 88.92.6, 88.92, 88.93, 88.94.1, 88.94.3, 88.95.1, 88.95.4, 88.95.7, 92.09.2, 92.09.8, 92.11.5, 92.18.14, 88.01.1, 88.01.5, 88.71.3, 88.91.1, 88.91.8, 88.92.2, 88.92.3, 88.92.5, 88.92.8, 92.03.4, 87.04.1, 87.11.4, 87.13.1, 87.17.3, 87.37.2, 87.42.1, 87.42.2, 87.42.3, 88.38.8, 88.42.1, 88.48, 88.63.2, 88.66.1, 88.83.1, 88.85, 88.95.3, 88.95.6, 87.13.2, 87.16.4, 87.76, 88.74.6, 88.74.7, 88.74.8, 88.74.9, 88.76.4, 92.20.6, 92.20.7, 87.04.2, 87.04.3, 87.04.4, 87.04.5, 87.04.6, 87.04.7, 87.04.8, 87.04.9, 87.08.1, 87.08.2, 87.08.3, 87.08.4, 87.08.5, 87.08.6, 87.08.7, 87.08.8, 87.09.5, 87.09.6, 87.09.7, 87.09.8, 87.09.9, 87.75.3, 87.75.4, 87.75.5, 88.01.9, 88.01.8, 87.41.2, 88.01.7, 87.41.3, 88.99.6, 88.77.4, 87.05, 87.06, 87.07, 87.35, 87.37.1, 87.37.3, 87.38, 87.52, 87.54.1, 87.59.1, 87.72, 87.73, 87.74.1, 87.74.2, 87.75.1, 87.76.1, 87.77, 87.79.1, 87.83, 88.32, 88.33.1, 88.38.2, 88.38.4, 88.38.7, 88.91.2, 88.92.4, 88.92.7, 88.92.9, 38.97.1, 38.97.2, 88.79.F, 87.42.4, 87.42.5, 87.42.6, 88.41.3, 88.43, 88.44.1, 88.45, 88.45.1, 88.47.1, 88.47.2, 88.47.3, 92.0531, 92.0532, 92.19.5, 92.0922, 88.38.B, 88.38.D, 88.38.E, 54.91.2, 87.0341, 88.27.2, 88.79.D, 92.1821, 88.7962, 87.1111, 92.19.4, 87.1121, 87.1122, 87.1112, 88.27.1, 87.4323, 87.89.1, 87.4311, 87.4321, 88.27.3, 88.3312, 88.3311, 87.24.2, 87.4322, 88.38.A, 34.91.1, 88.38.C, 88.48.1, 88.9433, 88.38.F, 88.9221, 88.41.1, 88.9571, 88.41.2, 92.1822, 87.4312, 87.24.1, 88.65.3, 88.66.2, 92.04.2, 92.04.3, 92.29.2, 92.29.5, 88.71.2, 88.72.A, 88.77.1, 88.76.A, 88.77.2, 88.74.A, 92.05.1, 92.11.1, 92.11.2, 87.03.3, 87.03.6, 87.41.1, 87.43.2, 87.71.1, 88.01.2, 88.01.4, 88.25, 88.42.2, 88.65.2, 88.75.A, 88.91.4, 88.92.1, 88.93.1, 88.94.2, 88.94.4, 88.95.2, 88.95.5, 88.97.7, 90.05.1, 88.97.4, 88.97.5, 88.97.6, 89.38.6, 92.19.9, 92.01.5, 92.11.8, 92.15.6, 92.16.2, 92.18.7, 92.18.8, 87.42.B, 88.29, 88.79.C, 88.91.9, 88.91.B, 88.91.C, 88.91.D, 88.91.E, 88.94.C, 88.95.9, 88.71.5, 88.95.8, 88.95.A, 88.95.B, 92.03.7, 92.05.7, 87.37.4, 87.37.5, 87.63.1, 88.42.3, 88.76.3, 88.78.A, 88.79.B, 88.91.A, 87.64.2, 8703, 87032, 87034, 87035, 87037, 87039, 87041, 87061, 87111, 87112, 87114, 87121, 87122, 87161, 87171, 87172, 8722, 8723, 8724, 8729, 8741, 87491, 8761, 8762, 87621, 87622, 87623, 8763, 8764, 87651, 87653, 8766, 87691, 8772, 8773, 8776, 8779, 88011, 88013, 88015, 8819, 8821, 8822, 8823, 8825, 8826, 8828, 88291, 88292, 8831, 88311, 88332, 88381, 88383, 88385, 88386, 88388, 88711, 88713, 88714, 88721, 88722, 88723, 88724, 88725, 88731, 88732, 88733, 88734, 88735, 8878, 88741, 88742, 88743, 88744, 88745, 88751, 88752, 88761, 88762, 88781, 88782, 88791, 88792, 88793, 88794, 88795, 88796, 88797, 88798, 88901, 88902, 88903, 88911, 88913, 88915, 88916, 88918, 8892, 88922, 88923, 88925, 88926, 88941, 88928, 8893, 8893A, 88943, 8894A, 9514, 88951, 88953, 88954, 88956, 92013, 92021, 92033, 92161, 92291, 9513, 87432CL, 87432CR, 87034I, 87034S, 8703B, 87111I, 87111S, 87112I, 87112S, 87432L, 87432R, 8821AL, 8821AR, 8821BL, 8821BR, 8821CL, 8821CR, 8822AL, 8822AR, 8822BL, 8822BR, 8823AL, 8823AR, 8823BL, 8823BR, 8826A, 8826BL, 8826BR, 8827AL, 8827AR, 8827BL, 8827BR, 8827CL, 8827CR, 8828AL, 8828AR, 8828BL, 8828BR, 88292L, 88292R, 88383AL, 88383AR, 88383BL, 88383BR, 88383CL, 88383CR, 88386AL, 88386AR, 88386BL, 88386BR, 88386CL, 88386CR, 88388AL, 88388AR, 88388BL, 88388BR, 88388CL, 88388CR, 88732L, 88732R, 88734L, 88734R, 92116, 92117, 92181, 92182, 88.99.4, 92.18.3, 3H11, 91.90.5, 57.17, 92.11.7, 38.94, 38.94.2, 92.09.9, 87.12.3, 88.79.E, 92.09.1, 92.11.6, 88.79.I, 88.79.J, 92.31.5, 92.30.2, 92.30.3, 87.79.2, 88.02.1, 88.25.1, 88.76.6, 90.93.2, 88.39.A, 88.28.2, 88.21.1, 88.26.1, 88.28.1, 88.79.G, 88.38.Z, 88.49.2, 87.69.4, 88.38.P, 88.39.G, 88.39.H, 87.03.D, 87.03.A, 88.38.Y, 88.79.H, 88.77.7, 87.16.6, 88.38.X, 88.39.8, 88.77.6, 87.16.7, 88.38.N, 87.03.E, 88.79.A, 87.03.C, 88.38.Q, 87.44.1, 88.76.5, 87.03.F, 87.43.5, 88.21.3, 88.77.5, 88.39.9, 88.38.9, 87.03.B, 92.29.B, 92.19.8, 50.91.1 |
| # of other instrumental tests in the last 6 months | Health-care system utilization | 0,1,2+ [0,0.5,1] |  |  |  | 89.5, 89.52, 89.54, 06.11.1, 6.13, 34.24, 40.11, 41.31, 49.23, 50.11, 50.19.1, 51.12, 54.22, 54.24.1, 83.21, 83.21.1, 85.11, 85.11.1, 86.11, 92.09.1, 92.09.2, 92.11.5, 92.11.6, 92.11.7, 92.18.6, 40.19.1, 40.19.2, 54.24, 88.99.2, 88.99.3, 88.99.4, 88.99.5, 42.24, 44.14, 45.14, 45.16, 45.24, 45.25, 45.29.2, 48.24, 89.65.1, 90.59.4, 90.83.3, 90.83.4, 90.83.5, 90.84.1, 90.84.2, 90.84.3, 90.84.4, 90.84.5, , 90.85.3, 90.85.5, 90.86.1, 90.86.2, 90.86.3, 90.86.4, 90.86.5, 90.87.1, 90.87.2, 90.87.3, 90.88.3, 90.88.5, 90.89.5, 90.90.5, 90.91.4, 90.93.1, 90.93.3, 90.93.4, 90.93.5, 90.94.1, 90.94.2, 90.94.3, 90.94.5, 90.95.1, 90.95.4, 91.01.3, 91.01.4, 91.01.5, 91.02.1, 91.02.2, 91.02.3, 91.03.4, 91.04.1, 91.05.3, 91.06.4, 91.08.4, 91.09.2, 91.11.2, 91.11.3, 91.11.5, 91.12.1, 91.12.2, 91.12.3, 91.12.4, 91.12.5, 91.13.1, 91.13.2, 91.14.1, 91.14.2, 91.14.3, 91.14.4, 91.14.5, 91.15.2, 91.15.3, 91.15.4, 91.15.5, 91.16.4, 91.16.5, 91.21.5, 91.24.1, 91.25.4, 91.26.4, 91.27.5, 91.33.4, 91.33.5, 91.34.1, 91.34.2, 91.34.3, 91.34.4, 91.34.5, 91.35.1, 91.35.3, 91.35.4, 91.35.5, 91.38.5, 91.39.1, 91.39.2, 91.39.3, 91.39.4, 91.39.5, 91.40.2, 91.40.3, 91.40.4, 91.40.5, 91.41.1, 91.41.2, 91.41.3, 91.41.4, 91.41.5, 91.42.3, 91.42.4, 91.42.5, 91.43.1, 91.43.2, 91.43.3, 91.43.4, 91.43.5, 91.44.1, 91.44.2, 91.44.3, 91.44.4, 91.44.5, 91.45.1, 91.45.2, 91.45.3, 91.45.4, 91.45.5, 91.46.1, 91.46.2, 91.46.3, 91.46.5, 91.47.1, 91.47.2, 91.47.3, 91.47.5, 91.48.1, 91.48.2, 91.48.4, 93.08.1, 93.08.2, 93.08.3, 04.11.1, 89.14, 89.14.1, 89.14.2, 89.14.3, 89.14.4, 89.14.5, 89.15.1, 89.15.2, 89.15.4, 89.15.8, 89.15.9, 89.19.1, 8.11, 9.11, 9.12, 10.21, 16.22, 95.23, 95.25, 24.11, 24.12, 25.01, 26.11, 27.21, 27.23, 67.12, 67.19.1, 68.16.1, 70.24, 70.29.1, 71.11, 18.12, 20.32.1, 21.22, 29.12, 31.43, 57.33, 58.23, 58.5, 60.11, 60.11.1, 60.13, 62.11, 64.11, 89.23, 33.24, 89.37.1, 89.37.2, 89.37.3, 89.37.4, 89.37.5, 89.37.6, 89.38.2, 89.65.2, 89.65.3, 89.65.6, MW1U, MW21, 90.90.1, 90.98.4, 89.43, 91.26.6, 91.15.1, 88.99.1, 89.15.3, 91.13.5, 91.16.3, 91.24.2, 91.26.3, 91.03.5, 91.21.1, MW_3924, MW_4256, MW_4274, MW_4275, MW_4277, MW_4278, 91.13.3, 45.23.2, 45.24.1, 45.25.1, 46.85, 58.60.3, 88.73.6, 88.73.7, 90.86.8, 90.88.6, 90.94.6, 92.19.8, 91.16.7, 91.15.7, MW_6307, MW_6308, 06.11.2, 44.19.3, 44.22.1, 44.43.1, 48.29.2, 52.19.1, 85.11.2, 87.24, 89.14.6, 89.54.4, 89.67.2, 90.48.5, 90.85.4, 90.88.4, 90.89.4, 90.90.4, 90.91.5, 90.92.1, 90.98.2, 90.98.3, 91.03.2, 91.03.3, 91.06.3, 91.07.4, 91.07.5, 91.08.3, 91.11.4, 91.13.4, 91.14.6, 91.17.4, 91.19.1, 91.19.2, 91.20.4, 91.23.1, 91.23.2, 91.23.3, 91.23.4, 91.23.5, 91.24.4, 91.24.5, 91.25.2, 91.25.3, 91.25.5, 91.26.1, 91.26.2, 91.26.5, 91.27.2, 91.27.3, 91.27.4, 91.27.7, 91.31.7, 91.42.1, 91.42.2, 92.18.10, 92.18.11, 92.18.12, 92.18.13, 92.18.7, 92.18.8, 92.18.9, 45.23, 48.23, 89.17, 91.19.3, 91.22.3, 91.17.1, 91.17.2, 91.17.5, 91.18.1, 91.18.3, 91.18.4, 91.18.5, 91.19.5, 91.20.1, 91.22.1, MW_4329, MW_4330, 45.42, 91.17.3, 91.18.2, 91.19.4, 91.20.2, 91.20.3, 91.20.5, 91.21.2, 91.21.3, 91.21.4, 91.22.2, 91.22.4, 91.22.5, 91.24.3, 91.25.1, 91.27.1, 91.35.2, 91.46.4, 42.33.1, 43.41.1, 45.13, 57.49.1, 89.7, 91.16.6, 88.80.1, 88.80.2, 88.80.3, 88.80.4, 44.19.4, 91.46.6, 91.46.7, 91.62.4, 91.61.6, 91.14.7, 91.14.8, 91.14.9, 91.15.6, 33.26, 38.21, 56.33, 77.4, 90.53.7, 89.45, 91.80.1, 51.10.1, 91.80.2, 45.13.3, 91.80.4, 91.80.5, 91.80.6, 45.13.4, 45.13.5, 51.11.1, 91.38.6, 92.11.8, 91.39.6, 92.20.9, 77.04.1, 77.04.2, 91.50.5, 91.50.6, 91.50.7, 91.50.8, 91.50.9, 91.51.1, 91.51.2, 91.51.3, 91.51.4, 91.51.5, 91.51.6, 91.51.7, 91.51.8, 91.51.9, 91.52.1, 91.52.2, 91.52.4, 91.53.2, 91.53.3, 91.53.4, 91.53.5, 91.53.6, 91.54.1, 91.54.2, 91.54.3, 91.54.4, 91.54.6, 91.54.7, 91.54.8, 91.54.9, 91.55.1, 91.55.2, 91.55.3, 91.55.4, 91.55.5, 91.55.6, 91.55.7, 91.55.8, 91.55.9, 91.56.1, 91.56.2, 91.56.3, 91.56.4, 91.56.5, 91.56.6, 91.56.7, 91.56.8, 91.56.9, 91.57.1, 91.57.2, 91.57.3, 91.57.4, 91.57.5, 91.57.7, 91.57.8, 91.57.9, 91.58.1, 91.58.6, 91.58.7, 91.58.8, 91.58.9, 91.59.1, 91.59.2, 91.59.3, 91.59.5, 91.59.6, 91.59.7, 91.59.8, 91.59.9, 91.71.1, 91.71.3, 91.71.4, 91.71.5, 91.71.8, 91.72.2, 91.72.3, 91.72.4, 91.72.6, 91.72.7, 91.72.8, 91.72.9, 91.73.1, 91.73.2, 91.73.3, 91.73.5, 91.73.6, 91.73.7, 91.73.8, 91.73.9, 91.74.1, 91.74.2, 91.74.3, 91.74.4, 91.74.5, 91.74.6, 91.74.7, 91.74.9, 91.75.1, 91.75.2, 91.75.3, 91.75.4, 91.75.5, 91.75.6, 91.75.7, 91.75.8, 91.76.1, 91.76.2, 91.76.3, 91.76.4, 91.76.5, 91.76.6, 91.77.1, 91.77.2, 11.22, 16.23, 22.11, 25.02, 27.22, 28.11, 44.15, 44.22, 48.26, 49.22, 55.23, 61.11, 67.11, 09.19.1, 1.1.1, 91.46.8, 9.19, 89.59.1, 92116, 92117, 92186, 93.08.4, 96.22, PC262, 91.2631, 91.2211, 91.1232, 91.1231, 91.20.6, 91.2214, 91.2216, 91.2215, 91.2361, 91.2362, 91.21.B, 91.23.7, 91.2373, 91.2372, 91.23.8, 91.2383, 91.2382, 91.25.6, 91.2632, 91.26.8, 91.26.7, 91.13.8, 90.90.3, 90.89.6, 91.15.A, 90.9544, 33.27, 34.23, 42.33.4, 43.11, 43.41.4, 89.66, 90.83.7, 91.01.A, 85.11.6, 45.14.2, 90.84.8, 42.93, 44.14.1, 45.26.2, 45.42.2, 48.24.1, 52.14, 45.19.2, 91.4641, 85.11.4, 91.4451, 91.4031, 91.4711, 91.4052, 91.4051, 91.4452, 90.98.5, 91.15.D, 91.18.6, 91.23.F, 91.2232, 45.42.1, 51.14.1, 4.12, 12.22, 12.22.1, 40.11.2, 42.24.1, 45.14.3, 45.16.1, 45.16.2, 45.23.3, 45.23.4, 45.24.2, 45.25.2, 45.25.3, 85.11.3, 85.11.5, 85.11.7, 85.11.9, 85.19.1, 93.08.F, 89.52.2, 89.52.3, 92.0911, 92.1972, 91.07.6, 90.93.7, 90.93.J, 90.93.K, 90.99.1, 91.12.8, 91.17.6, 91.21.6, 91.24.8, 91.24.F, 91.24.G, 91.25.8, 91.25.D, 91.26.C, 91.15.F, 98.05, 98.03, 98.12, 57.39.2, 89.38.1, 92.0922, 33.24.1, 92.1861, 92.1981, 92.0921, 45.14.1, 92.1971, 52.13, 89.52.1, 91.38.A, 91.41.6, 91.2218, 91.08.7, 90.99.4, 91.17.8, 91.26.9, 90.9551, 48.23.1, 91.0313, 91.13.7, 90.88.7, 91.19.6, 91.20.7, 91.26.E, 22.9, 68.12.1, 6.01, 11.21, 31.42, 50.91, 89.15.5, 90.96.2, 91.04.3, 89.41, 91.04.5, 90.95.3, 90.90.2, 91.37.1, 34.91.1, 90.23.2, 91.10.1, 91.29.4, 91.29.5, 90.83.6, 90.85.1, 90.85.2, 90.85.6, 90.87.6, 90.91.6, 90.95.8, 90.97.3, 91.01.2, 91.01.8, 91.02.4, 91.02.6, 91.02.7, 91.02.8, 91.03.6, 91.16.1, 91.16.2, 91.39.7, 91.40.1, 33.26.1, 52.11.1, 52.11.2, 54.23, 68.16.2, 77.40.1, 49.31, 42.33.3, 43.41.3, 45.43.2, 52.13.1, 52.14.1, 45.13.B, 45.23.B, 87.16.1, MAC12, 9.43, 45.17, 45.26.1, 9068101, 9083323, 9087428, 9087431, 9087435, 9087462, 9112607, 9112610, 9112622, 9112626, 9143101, 90.31.4, 9083305, 9083309, 9083311, 9083332, 9087430, 9087447, 9087456, 91.36.4, 9112601, 9112602, 9112619, 9112623, 9128201, 9141301, 92.19.7, 70.21, R25.02, R66.2, R66.21, R66.22, R67.11, R85.12, 45.23.1, 89.44.1, 90.85.7, 90.86.6, 90.86.7, 90.93.9, 90.94.7, 90.96.6, 90.92.6, 90.93.8, 91.12.6, 91.12.7, 91.22.6, 91.22.7, 91.23.6, 91.24.6, 91.25.7, 91.21.7, 91.38.9, 92.05.8, 92.19.9, 99.3, 45.13.1, 51.12.1, 88.79.A, 88.99.6, 89.54.1, 89.54.2, 89.54.3, 89.54.5, 89.48.2, 88991, 88995, 4111, 6111, 811, 2611, 3424, 4011, 40193, 40194, 4224, 4513, 4516, 4523, 4524, 4525, 4542, 4824, 4923, 50191, 5112, 5422, 54241, 5631, 5732, 5733, 5823, 58603, 6211, 7024, 8914, 89153, 89374, 8941, 8943, 8944, 8950, 8954, 89611, 93081, PCA18, PCA28, PCA87, PCA88, PCA0C, PCA0D, 90833, 90834, 90843, 90844, 90845, 90853, 90854, 90855, 90865, 90873, 90895, 90901, 90933, 90934, 90935, 90942, 90943, 90954, 90982, 90984, 91014, 91021, 91022, 91023, 91035, 91064, 91115, 91121, 91133, 91134, 91135, 91145, 91151, 91152, 91155, 91164, 91165, 91212, 91221, 91242, 91262, 91265, 91273, 91392, 91393, 91395, 91402, 91405, 91411, 91415, 91421, 91422, 91423, 91424, 91425, 91433, 91434, 91435, 91451, 91452, 91454, 91463, 91465, 91471, 91472, 91473, 4514, 6011, 89145, 89371, 89372, 8952, 92091, 92092, 90931, 91131, 91143, 91154, 91163, 91173, 91181, 91204, 91211, 91222, 91223, 91225, 91231, 91234, 91235, 91245, 91253, 91404, 91412, 91413, 91414, 91431, 91432, 91441, 91442, 91443, 91444, 91445, 91453, 91455, 91461, 91462, 91464, 91475, 91481, 91482, 90885, 4513BB, 4523B, 4524B, 91221C, 6112, 5011, 60111, 88992A, 88992B, 88992C, 89158, 90872, 91211EA, 91211EB, 91211VC, 91232, 91264G, 91264M, PACC261, PACC262, PACC348B, PACC560, PACC561, 88993, 91141, 91.39.D, 67.29.1, 91.39.L, 91.39.M, 90.69.7, 90.69.8, 90.70.2, 91.39.N, 91.05.7, 91.11.6, 91.41.A, 91.39.J, 91.39.K, 91.39.A, 91.39.B, 91.39.C, 91.39.E, 91.39.F, 91.39.G, 91.39.H, 91.40.A, 91.40.B, 91.40.C, 91.40.E, 91.40.F, 91.40.H, 91.41.B, 91.41.C, 91.41.F, 91.41.G, 91.41.K, 91.42.A, 91.42.D, 91.42.E, 91.42.G, 91.42.H, 91.43.A, 91.43.C, 91.43.F, 91.43.G, 91.43.H, 91.43.J, 90.49.7, 91.06.9, 91.30.3, 91.06.8, 91.06.1, 91.39.P, 92.09.3, 92.15.5, 92.03.5, 92.14.3, 91.41.J, 91.42.C, 87.66, 33.26.2, 42.33.2, 43.41.2, 45.29.5, 45.43.1, 51.16, 77.44, 91.38.7, 91.39.9, 97.32, 89.59.2, 42.33, 46.32, 99.29.A, 92.09.8, 89.61.1, 91.49.3, 90.92.3, 91.06.6, 91.27.6, 91.08.6, 91.27.8, 91.47.4, 303210016, 618211930, 618218360, 618218390, 618218420, 1224246030, 87.54.1, 55.92, 89.15.6, 89.44, 91.07.9, 91.24.B, 91.24.C, 91.38.F, 91.38.G, 91.38.H, 85.11.8, 91.47.E, 91.47.G, 91.47.H, 91.47.I, 91.42.7, 91.41.8, 91.45.9, 90.83.8, 93.94, 91.41.9, 88.99.7, 88.99.8, 91.45.7, 90.93.6, 91.45.6, 91.24.A |
| Any surgery in the last 6 months | Health-care system utilization | 0,1 [0,1] |  |  |  | 48.35, 49.31, 49.39, 49.46, 83.31, 85.21, , 86.4, 64.2, 70.33.1, 71.31, 42.33.1, 42.33.2, 43.41.1, 43.41.2, 45.43.1, 91.47.4, 8.21, 8.22, 9.21, 9.6, 10.31, 11.32, 11.39, 23.3, 24.31, 24.4, 27.49.1, 76.2, 76.77, 04.07.1, 79, 79.01, 79.02, 79.03, 79.04, 79.07, 79.08, 93.51, 93.52, 93.53, 93.54.2, 93.54.5, 93.54.6, 93.54.8, 97.1, 97.88, 68.29.1, 18.29, 21.31, 21.71, 58.31, MW31, MW7I, 18.21, 18.31, 68.2, 71.30.1, MW_4310, MW_4311, MW_4336, MW_6857, 26, 33.24, 86.30.4, 91.53.9, 91.57.6, 91.58.4, 91.58.5, 91.59.4, 91.71.2, 21.32, 45.3, 58.39, 67.39, 86.3, 67.32, 67.33, PR10, PR17, PR18, PR4A, PR4B, PR4C, PR4E, PR4F, PR11A, PR11B, PR11C, PR11D, PR11E, 45.30.2, 49.04, 18.31.1, 23.50.1, 27.31, 27.43, 38.53, 49.11, 79.3, 10.33, 45.43.2, 11.31, 90.52.5, 86.4.7, 23.09, 23.19, 86.30.3, 83.09, 38.69, 82.39, 49.39.1, 82.21, 82.29, 80.61, 93.54.4, 93.54.7, R04.07, R09.6, R12.42, R13.65, R18.21, R18.31, R18.39, R21.62, R25.1, R27.31, R27.42, R27.43, R27.72, R38.69, R45.33, R45.41, R49.04, R61.2, R62.2, R63.1, R63.2, R63.3, R66.61, R67.39, R71.21, R71.24, R71.3, R77.63, R77.64, R77.66, R77.67, R80.6, R80.81, R80.82, R80.86, R82.21, R82.29, R83.32, R83.39, R83.49, R86.0, R86.3, R86.91, 45.13, 69.1, 86.30.0, 90.19.6, 821, 822, 1031, 1829, 2131, 2431, 27491, 42332, 43411, 43412, 45431, 4939, 5831, 642, 6732, 6733, 68291, 71301, 762, 7677, 7900, 7902, 7903, 7904, 7907, 7908, 864, 93545, 93548, 91474, 86303, 86.30.1, 12.42, 12.44, 49.46.1, 63.1, 63.2, 68.29.3, 71.24, 77.6, 77.65, 77.66, 77.67, 77.68, 82.22, 86.21, 91.40.D, 91.41.F, 91.41.H, 733.82, 805, 808, 810, 811, 812, 813, 814, 815, 816, 823, 824, 825, 826, 820 - 821, 16.92, 77.51, 77.52, 77.53, 77.59, 80.98, 91.40.5, 603090280, 603090350, 603150330, 603180090, 603185330, 606300060, 606306020, 612030540, 612030690, 612060180, 612090270, 612120510, 612186350, 618156050, 618156080, 618156110, 618156290, 618215690, 618215720, 618215750, 618217760, 618218090, 618218300, 618218310, 624096280, 624096340, 624096430, 624096460, 624185150, 624185180, 624215900, 624215930, 624215960, 624218510, 624218540, 1224031630, 99.99.9, 86.02.1, DRG57834, 86.30.5 |
| # blood exams prescribed in the last 6 months | Health-care system utilization | 0,1,2+ [0,0.5,1] | [software’s internal codes] | | | |
| Any blood tumoral marker prescribed in the last 6 months | Health-care system utilization | 0,1 [0,1] | [software’s internal codes] | | | |
| Geriatric evaluation prescribed in the last 6 months | Health-care system utilization | 0,1 [0,1] | [software’s internal codes] | | | |
| # of ambulatory specialist visit in the last 6 months | Health-care system utilization | 0,1,2+ [0,0.5,1] | [software’s internal codes] | | | |
| Palliative-care evaluation prescribed in the last 6 months | Health-care system utilization | 0,1 [0,1] | [software’s internal codes] | | | |
| Emergency department visit in the last 6 months | Health-care system utilization | 0,1 [0,1] | [software’s internal codes] | | | |
| Hospital admission in the last 6 months | Health-care system utilization | 0,1 [0,1] | [software’s internal codes] | | | |
| Other non-specific findings in the last 6 months | signs/symptoms | 0,1 [0,1] | 790,791 ,792, 793, 794, 795, 796 |  |  |  |
| Pain in the last 6 months | signs/symptoms | 0,1 [0,1] | 338.4 |  | M01AB, M01AC , M01AE , M01AH , N02A, N02BE01 |  |
| Confusional state in the last 6 months | signs/symptoms | 0,1 [0,1] | 780.02, 780.09, 780.97 |  |  |  |
| Syncope or dizziness in the last 6 months | signs/symptoms | 0,1 [0,1] | 780.2, 780.4 |  |  |  |
| Changes in taste or smell in the last 6 months | signs/symptoms | 0,1 [0,1] | 781.1 |  |  |  |
| Tachycardia in the last 6 months | signs/symptoms | 0,1 [0,1] | 785.0, 785.1 |  |  |  |
| Cough in the last 6 months | signs/symptoms | 0,1 [0,1] | 786.2 |  |  |  |
| Fatigue or malaise in the last 6 months | signs/symptoms | 0,1 [0,1] | 780.7 |  |  |  |
| Dyspnoea in the last 6 months | signs/symptoms | 0,1 [0,1] | 786.0, 786.7 |  |  |  |
| Edema in the last 6 months | signs/symptoms | 0,1 [0,1] | 782.3 |  |  |  |
| Chest pain in the last 6 months | signs/symptoms | 0,1 [0,1] | 786.5 |  |  |  |
| Fever in the last 6 months | signs/symptoms | 0,1 [0,1] | 780.6 |  |  |  |
| Diarrhea in the last 6 months | signs/symptoms | 0,1 [0,1] | 787.91 |  |  |  |
| Stipsis or constipation in the last 6 months | signs/symptoms | 0,1 [0,1] | 564 |  | A06A |  |
| Nausea or vomiting in the last 6 months | signs/symptoms | 0,1 [0,1] | 787 |  |  |  |
| Gastroenteric symptoms in the last 6 months | signs/symptoms | 0,1 [0,1] | 787.0, 564, 787.2, 787.1, 789.0, 787.91, 787.63, 787.7 |  | A06A |  |
| Falls | signs/symptoms | 0,1 [0,1] | E880, E885, E886 |  |  |  |
| Mild/moderate functional limitations | socio/economic/functional condition | 0,1 [0,1] |  | L01, L02, CO1, CO3, L03, L04, B12, A04 |  |  |
| Severe functional limitation | socio/economic/functional condition | 0,1 [0,1] |  | CO2, A08 |  |  |
| Financial difficulties | socio/economic/functional condition | 0,1 [0,1] |  | RE01 ,RE02 , RE03 , RE04, RE1, RE2, RE3, RE4, RE14, RE35, RED, REI, |  |  |

**Table S3.** Operationalization of the 25 deficits selected for the Primary Care Frailty Index (PC-FI) in SNAC-K.

| **Deficit** | **Definition** |
| --- | --- |
| Dementia or cognitive decline | Dementia diagnosis (ICD-10 codes, ATC codes, medical history, regional and national registries, physical and instrumental exams – as described in the supplementary material of [10.1093/gerona/glw233](https://dx-doi-org.proxy.unibs.it/10.1093%2Fgerona%2Fglw233)) or Minimental State Examination Score < 24 |
| Severe disability | Nurse questionnaire and evaluation of daily activities of daily living (1+ ADL limitation) |
| Cerebrovascular diseases | ICD-10 codes, ATC codes, medical history, regional and national registries, physical and instrumental exams – as described in the supplementary material of [10.1093/gerona/glw233](https://dx-doi-org.proxy.unibs.it/10.1093%2Fgerona%2Fglw233) |
| Solid neoplasms | ICD-10 codes, ATC codes, medical history, regional and national registries, physical and instrumental exams – as described in the supplementary material of [10.1093/gerona/glw233](https://dx-doi-org.proxy.unibs.it/10.1093%2Fgerona%2Fglw233) |
| COPD/emphysema | ICD-10 codes, ATC codes, medical history, regional and national registries, physical and instrumental exams – as described in the supplementary material of [10.1093/gerona/glw233](https://dx-doi-org.proxy.unibs.it/10.1093%2Fgerona%2Fglw233) |
| Ischemic heart disease | ICD-10 codes, ATC codes, medical history, regional and national registries, physical and instrumental exams – as described in the supplementary material of [10.1093/gerona/glw233](https://dx-doi-org.proxy.unibs.it/10.1093%2Fgerona%2Fglw233) |
| Heart failure | ICD-10 codes, ATC codes, medical history, regional and national registries, physical and instrumental exams – as described in the supplementary material of [10.1093/gerona/glw233](https://dx-doi-org.proxy.unibs.it/10.1093%2Fgerona%2Fglw233) |
| Chronic kidney disease | ICD-10 codes, ATC codes, medical history, regional and national registries, physical and instrumental exams – as described in the supplementary material of [10.1093/gerona/glw233](https://dx-doi-org.proxy.unibs.it/10.1093%2Fgerona%2Fglw233) |
| Atrial Fibrillation | ICD-10 codes, ATC codes, medical history, regional and national registries, physical and instrumental exams – as described in the supplementary material of [10.1093/gerona/glw233](https://dx-doi-org.proxy.unibs.it/10.1093%2Fgerona%2Fglw233) |
| Parkinson disease and parkinsonism | ICD-10 codes, ATC codes, medical history, regional and national registries, physical and instrumental exams – as described in the supplementary material of [10.1093/gerona/glw233](https://dx-doi-org.proxy.unibs.it/10.1093%2Fgerona%2Fglw233) |
| Previous hip fracture | Interview and physician’s evaluation of medical history |
| Anemia | ICD-10 codes, ATC codes, medical history, regional and national registries, physical and instrumental exams – as described in the supplementary material of [10.1093/gerona/glw233](https://dx-doi-org.proxy.unibs.it/10.1093%2Fgerona%2Fglw233) |
| Financial difficulties | Defined as the lack of financial assets to cover unforeseen health expenses |
| Hospital admission in the last 6 months | Hospital admission within 6 months from national registries |
| Chronic ulcers of the skin | ICD-10 codes, ATC codes, medical history, regional and national registries, physical and instrumental exams – as described in the supplementary material of [10.1093/gerona/glw233](https://dx-doi-org.proxy.unibs.it/10.1093%2Fgerona%2Fglw233) |
| Bradycardias or other cardiac conduction abnormalities | ICD-10 codes, ATC codes, medical history, regional and national registries, physical and instrumental exams – as described in the supplementary material of [10.1093/gerona/glw233](https://dx-doi-org.proxy.unibs.it/10.1093%2Fgerona%2Fglw233) |
| Other neurological diseases | ICD-10 codes, ATC codes, medical history, regional and national registries, physical and instrumental exams – as described in the supplementary material of [10.1093/gerona/glw233](https://dx-doi-org.proxy.unibs.it/10.1093%2Fgerona%2Fglw233) |
| Stypsis or constipation in the last 6 months | Physician interview |
| Any new low-molecular weight heparin prescription in the last 6 months | ATC code from current list of prescribed medication |
| peripheral vascular disease | ICD-10 codes, ATC codes, medical history, regional and national registries, physical and instrumental exams – as described in the supplementary material of [10.1093/gerona/glw233](https://dx-doi-org.proxy.unibs.it/10.1093%2Fgerona%2Fglw233) |
| Nutritional issue | Dysphagia (from physician interview and medical history) or malnutrition, defined as BMI < 18 |
| Diabetes | ICD-10 codes, ATC codes, medical history, regional and national registries, physical and instrumental exams – as described in the supplementary material of [10.1093/gerona/glw233](https://dx-doi-org.proxy.unibs.it/10.1093%2Fgerona%2Fglw233) |
| Schizophrenia and other delusional disorders | ICD-10 codes, ATC codes, medical history, regional and national registries, physical and instrumental exams – as described in the supplementary material of [10.1093/gerona/glw233](https://dx-doi-org.proxy.unibs.it/10.1093%2Fgerona%2Fglw233) |
| Edema in the last 6 months | Physical examination by physician |

**Table S4.** Discriminative ability (c-statistics) of unadjusted cox regression model in the prediction of mortality (maximum follow-up) in the whole population and among persons younger and older than 70 years old, calculated for the PC-FI and for individual deficits.

| Deficit | Whole sample  c-statistics | ≤ 70 years old  c-statistics | > 70 years old  c-statistics |
| --- | --- | --- | --- |
| *PC-FI* | *0.732* | *0.680* | *0.679* |
| Dementia or cognitive decline | 0.584 | 0.532 | 0.580 |
| Anemia | 0.570 | 0.531 | 0.566 |
| Ischemic heart disease | 0.567 | 0.540 | 0.554 |
| Financial difficulties | 0.564 | 0.526 | 0.510 |
| COPD/emphysema | 0.557 | 0.557 | 0.544 |
| Cerebrovascular diseases | 0.553 | 0.520 | 0.545 |
| Severe disability | 0.551 | 0.547 | 0.545 |
| Diabetes | 0.546 | 0.564 | 0.533 |
| Chronic kidney disease | 0.545 | 0.529 | 0.538 |
| Heart failure | 0.540 | 0.520 | 0.539 |
| Solid neoplasms | 0.538 | 0.572 | 0.525 |
| Atrial Fibrillation | 0.534 | 0.509 | 0.529 |
| Bradycardias or other cardiac conduction abnormalities | 0.523 | 0.508 | 0.520 |
| Parkinson disease and parkinsonism | 0.522 | 0.507 | 0.520 |
| Any new low-molecular weight heparin prescription in the last 6 months | 0.521 | 0.518 | 0.518 |
| Hospital admission in the last 6 months | 0.519 | 0.530 | 0.515 |
| Previous hip fracture | 0.518 | 0.502 | 0.518 |
| peripheral vascular disease | 0.512 | 0.511 | 0.510 |
| Chronic ulcers of the skin Chronic ulcers of the skin | 0.510 | 0.504 | 0.509 |
| Stypsis or constipation in the last 6 months | 0.510 | 0.510 | 0.508 |
| Any new oxygen prescription in the last 6 months | 0.508 | 0.506 | 0.508 |
| Other neurological diseases | 0.505 | 0.505 | 0.504 |
| Nutritional issues | 0.504 | 0.501 | 0.504 |
| Schizophrenia and other delusional disorders | 0.502 | 0.507 | 0.501 |
| Edema | 0.501 | 0.501 | 0.501 |

Abbreviations: COPD: chronic obstructive pulmonary disease; PC-FI: Primary Care Frailty Index

**Table S5.** Number and proportion of individuals in different frailty groups, stratified by age and sex in the HSD. Frailty proportions are reported within sex groups.

| Age groups (years) | Males  N=137,574 (44.6) | | Females  N=170,706 (55.4) | |
| --- | --- | --- | --- | --- |
| 60-69 N = 148975 (48.3) | Fit | 49688 (68.4) | Fit | 52636 (68.9) |
|  | Mild frailty | 18483 (25.5) | Mild frailty | 20019 (26.2) |
|  | Moderate frailty | 3628 (5.0) | Moderate frailty | 3196 (4.2) |
|  | Severe frailty | 806 (1.1) | Severe frailty | 519 (0.7) |
| 70-79  N = 102057 (33.1) | Fit | 16864 (37.5) | Fit | 24148 (42.3) |
|  | Mild frailty | 191977 (42.6) | Mild frailty | 24094 (42.3) |
|  | Moderate frailty | 6751 (15.0) | Moderate frailty | 6853 (12.0) |
|  | Severe frailty | 2196 (4.9) | Severe frailty | 1954 (3.4) |
| 80-89 N = 49803 (16.2) | Fit | 4089 (22.8) | Fit | 8629 (27.1) |
|  | Mild frailty | 7282 (40.6) | Mild frailty | 13587 (42.7) |
|  | Moderate frailty | 4411 (24.5) | Moderate frailty | 6744 (21.1) |
|  | Severe frailty | 2168 (12.1) | Severe frailty | 2893 (9.1) |
| 90+  N = 7445 (2.4) | Fit | 396 (19.7) | Fit | 1181 (21.7) |
|  | Mild frailty | 711 (35.4) | Mild frailty | 2092 (38.5) |
|  | Moderate frailty | 567 (28.2) | Moderate frailty | 1449 (26.7) |
|  | Severe frailty | 337 (16.7) | Severe frailty | 712 (13.1) |

Abbreviations: HSD: Health Search Database

**Table S6.** Psychometric properties of the PC-FI in the Health Search Database (HSD) for the prediction of mortality over the whole follow-up.

| PC-FI  Cut-off | % | Accuracy | Sensitivity | Specificity | PPV | NPV | LR+ | LR- |
| --- | --- | --- | --- | --- | --- | --- | --- | --- |
| ≥0.07 | 48.8 | 0.59 | 0.79 | 0.56 | 0.21 | 0.95 | 1.78 | 0.38 |
| ≥0.14 | 14.7 | 0.83 | 0.42 | 0.89 | 0.37 | 0.91 | 3.94 | 0.65 |
| ≥0.21 | 3.7 | 0.87 | 0.15 | 0.98 | 0.53 | 0.88 | 7.49 | 0.87 |

Abbreviations = PPV: positive predictive value, NPV = negative predictive value, LR+: positive likelihood ratio, LR-: negative likelihood ratio

**Figure S1**. Primary Care Frailty Index (PC-FI) distribution in the development and validation datasets by age and sex.


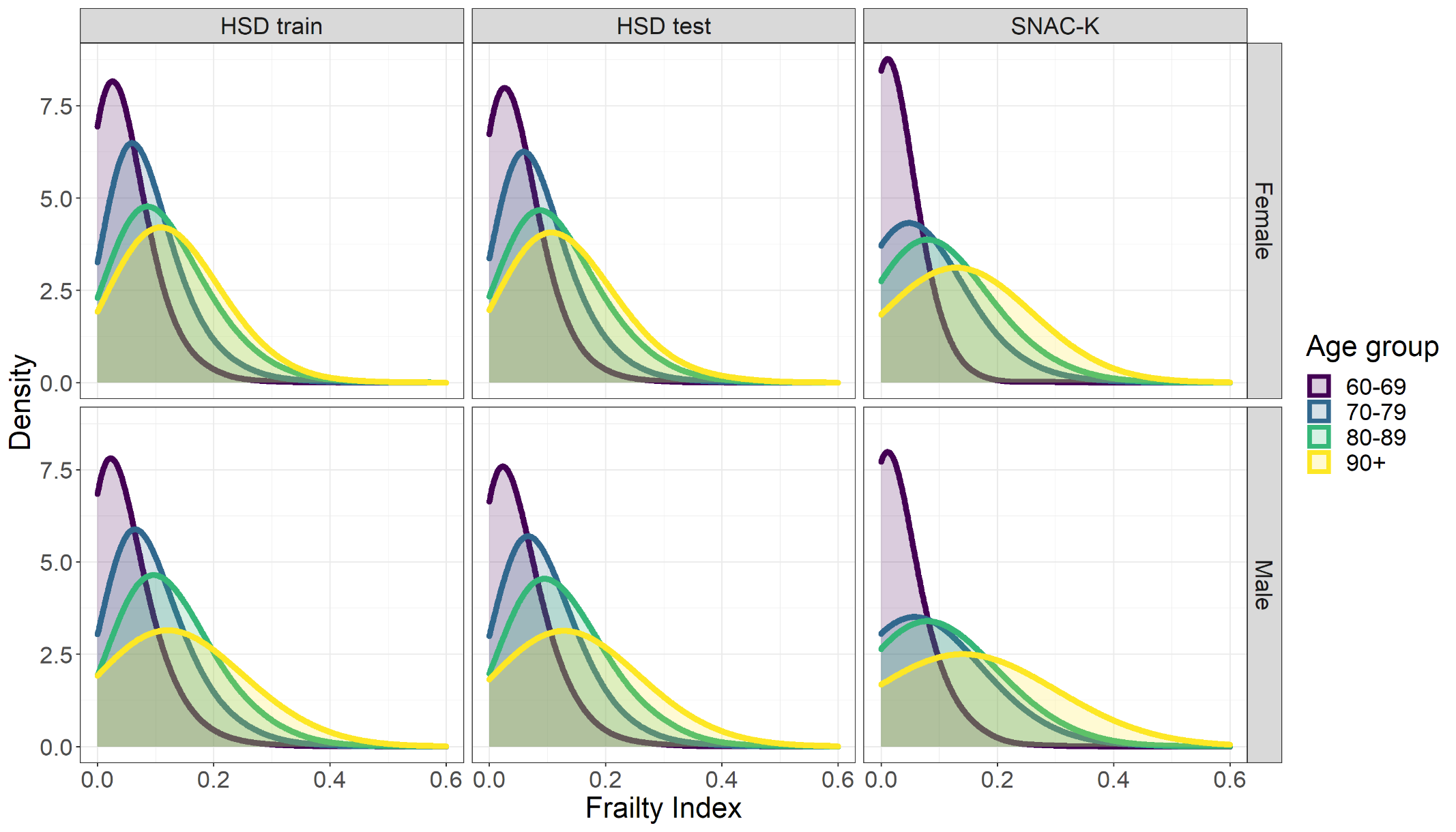


PC-FI score

Abbreviations: HSD: Health Search Database; SNAC-K: Swedish National Study on Aging and Care in Kungsholmen

**Figure S2.** Kaplan Maier survival curve across different frailty groups in the HSD.


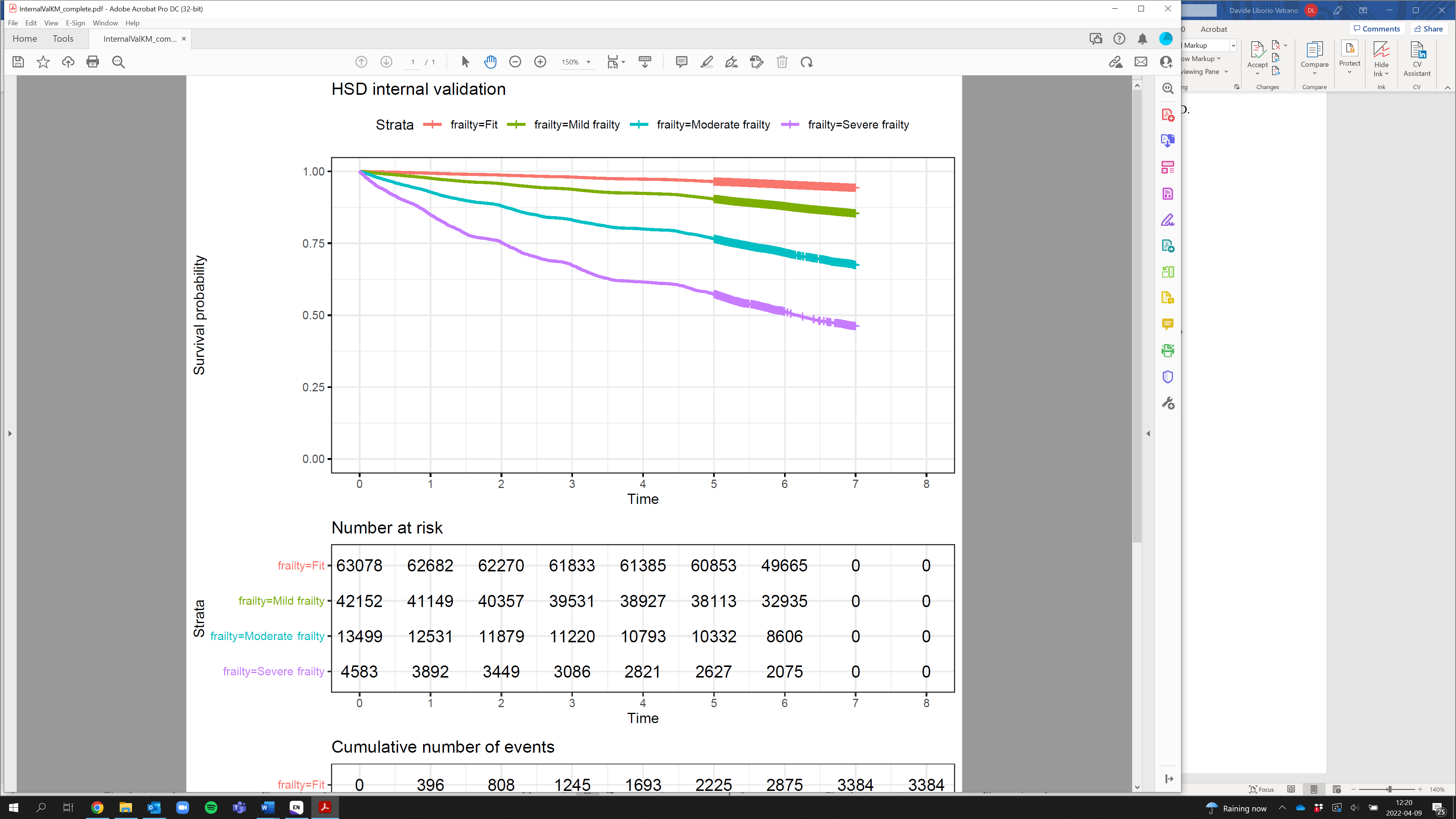


Abbreviations: HSD: Health Search Database

**Figure S3.** Reliability plot of the PC-FI for the prediction of mortality over the whole follow-up.


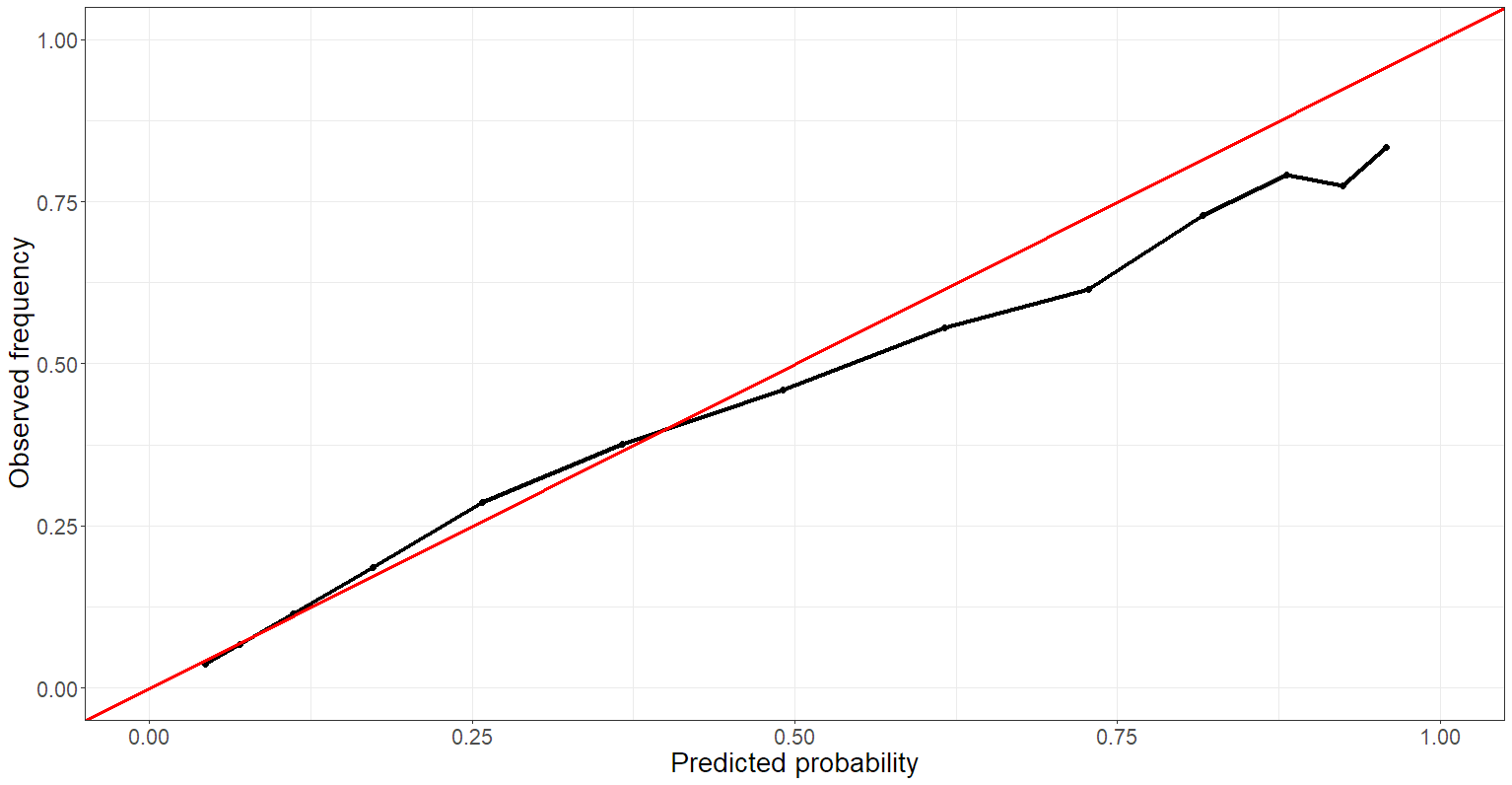


An unadjusted logistic regression model was fitted in the internal validation subsample, using mortality (over the whole follow-up period) as outcome and PC-FI as independent variable. The AUC of the model was 0.746. The plot shows high reliability of the model, with a slight pessimistic tendency for higher observed mortality frequency.
